# Development and Radiation Response Assessment in A Novel Syngeneic Mouse Model of Tongue Cancer: 2D Culture, 3D Organoids and Orthotopic Allografts

**DOI:** 10.3390/cancers12030579

**Published:** 2020-03-02

**Authors:** Vui King Vincent-Chong, Mukund Seshadri

**Affiliations:** 1 Department of Oral Oncology, Roswell Park Comprehensive Cancer Center, Buffalo, NY 14263, USA; Vincent.ChongVuiKing@RoswellPark.org; 2 Department of Dentistry and Maxillofacial Prosthetics, Roswell Park Comprehensive Cancer Center, Buffalo, NY 14263, USA

**Keywords:** oral cancer, squamous cell carcinoma, radiation therapy, organoids, orthotopic OSCC, magnetic resonance imaging

## Abstract

Oral squamous cell carcinoma (OSCC) are aggressive cancers that contribute to significant morbidity and mortality in humans. Although numerous human xenograft models of OSCC have been developed, only a few syngeneic models of OSCC exist. Here, we report on a novel murine model of OSCC, RP-MOC1, derived from a tongue tumor in a C57Bl/6 mouse exposed to the carcinogen 4-nitroquinoline-1-oxide. Phenotypic characterization and credentialing (STR profiling, exome sequencing) of RP-MOC1 cells was performed in vitro. Radiosensitivity was evaluated in 2D culture, 3D organoids, and in vivo using orthotopic allografts. RP-MOC1 cells exhibited a stable epithelial phenotype with proliferative, migratory and invasive properties. Exome sequencing identified several mutations commonly found in OSCC patients. The LD_50_ for RP-MOC1 cells in 2D culture and 3D organoids was found to be 2.4 Gy and 12.6 Gy, respectively. Orthotopic RP-MOC1 tumors were pan-cytokeratin+ and Ki-67+. Magnetic resonance imaging of orthotopic RP-MOC1 tumors established in immunocompetent mice revealed marked growth inhibition following 10 Gy and 15 Gy fractionated radiation regimens. This radiation response was completely abolished in tumors established in immunodeficient mice. This novel syngeneic model of OSCC can serve as a valuable platform for the evaluation of combination strategies to enhance radiation response against this deadly disease.

## 1. Introduction

Oral cancers are a major cause of morbidity and mortality worldwide with more than 90% of these cancers diagnosed histologically as oral squamous cell carcinoma (OSCC) [1]. Globally, approximately 350,000 of patients are diagnosed with OSCC resulting in more than 150,000 deaths every year [2]. Chronic exposure of the oral epithelium to carcinogens such as alcohol and tobacco are the major risk factors for development of OSCC [1,2,3]. Radiation therapy is an integral part of the management of patients with OSCC [4]. Significant advancements in radiation delivery methods have led to improved outcomes in patients but resistance to radiation remains a clinical problem [5,6].

The development of clinically relevant animal models of OSCC is an essential first step in the examination of novel strategies that could enhance radiotherapeutic efficacy against this debilitating disease. In this regard, numerous human tumor xenograft models of OSCC have been employed to examine the effects of radiotherapy in vivo [7,8,9]. However, the need for an immunodeficient host limits the utility of these models for radiation studies. While genetically engineered mouse models are useful in delineating the role of specific drivers of oral carcinogenesis, these models can exhibit variable penetrance and disease kinetics [10,11]. Alternatively, rodent models of OSCC based on chronic exposure of animals to chemical carcinogens such as 4-nitroquinoline-oxide (4NQO) have been developed and utilized for experimental studies [12,13]. 4NQO is a water-soluble chemical that leads to the malignant transformation and development of invasive cancer through intracellular oxidative stress and the formation of DNA adducts [14,15]. Studies by us and others have shown that 4NQO-induced tumors share histopathologic and molecular features of human OSCC and the model can serve as a useful platform to investigate the activity of chemopreventive agents against the spectrum of oral carcinogenesis [16,17,18]. Although disease progression in this model can be accelerated by simultaneous treatment with tumor promoters (e.g., ethanol), the requirement of chronic administration of the carcinogen (16–26 weeks) limits the widespread use of this model for therapeutic studies [19]. Considering the time-consuming nature of carcinogen-induced models and the paucity of immunocompetent murine models of OSCC, there has been increased interest in developing immunocompetent mouse models of OSCC. Nagaya et al. have previously developed murine OSCC models with varying immunogenicity to demonstrate the therapeutic potential of photoimmunotherapy targeting the stem cell marker CD44 [20]. Recently, Chen et al. have recently developed and examined the response of murine OSCC lines to chemotherapy and immune therapy [21,22]. Despite the well-documented two-way interaction between the immune system and radiation therapy [23,24,25], only a few studies have examined the effects of radiation against OSCC in immunocompetent hosts [26,27]. Given the paucity of syngeneic models of OSCC and the limited literature on the radiation response in these models, in the present study, we developed and credentialed the radiation response of a novel murine model of OSCC, RP-MOC1, derived from a tongue tumor in a C57BL/6NCr mouse exposed to 4NQO. The response of RP-MOC1 to radiation was evaluated in 2D cell culture, 3D organoids and in vivo using orthotopic allografts established in immunocompetent and immunodeficient hosts. Our results highlight the potential utility of this model as a platform for evaluation of combination strategies to enhance radiation response against OSCC.

## 2. Results

### 2.1. Generation and Credentialing of RP-MOC1

**Figure 1:**
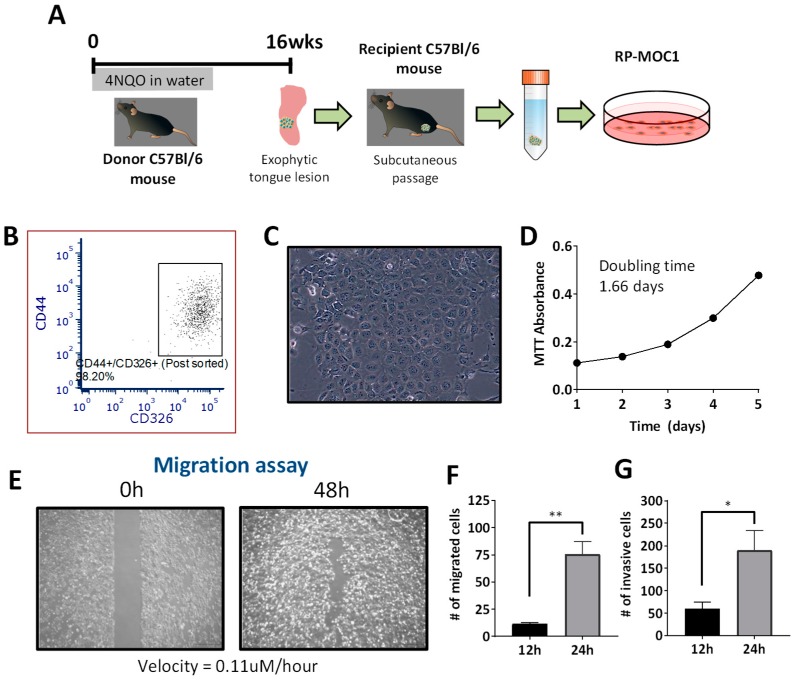
Establishment and in vitro behavior of the RP-MOC1 OSCC model. (**A**) Schematic shows workflow for generation of the RP-MOC1 cell line. Donor tongue tissue from a C57BL/6NCr mouse exposed to 4NQO in the drinking water for 16 weeks was initially transplanted subcutaneously to establish an allograft. The established tumor was subsequently excised, digested with collagenase and seeded in a petri dish. (**B**) Flow cytometry was used to sort cells co-expressing the stem cell marker (CD44) and epithelial cell adhesion molecule, EpCAM (CD326). (**C**) Microscopic image of RP-MOC1 showing characteristic cobblestone morphology with anisocytosis and cellular pleomorphism. Image was acquired at 20× magnification. (**D**) Growth curve of RP-MOC1 cells over a 5-day period. The doubling time for this cell line based on MTT was determined to be 37.6 ± 2.4 h. (**E**) Wound healing assay. Images of RP-MOC1 cells at 0 h and at 48 h are shown. The image at 48 h illustrates near complete wound closure due to migration of tumor cells. Images were acquired at 4× magnification. Scale bar: 500 μm. Bar graphs showing increase in the number of migrated cells (**F**) and invasive cells (**G**) between 12 h to 24 h. Data is reported as mean ± SEM and from two independent experiments. (ns, no significance; * *p* < 0.05; ** *p* < 0.01).

The workflow for generation of RP-MOC1 cells is shown schematically in Figure 1A. A clinically visible exophytic lesion on the tongue of a C57Bl/6 mouse exposed to the carcinogen 4NQO in drinking water for 16 weeks was transplanted subcutaneously into a recipient mouse. The established tumor was excised, and a portion of the tumor was also placed in formalin fixative for histopathologic evaluation. The remaining tissue was digested and seeded as a monolayer. Cultured cells were sorted using flow cytometry based on expression of the stem cell marker (CD44) and epithelial cell adhesion molecule, EpCAM (CD326). Post sort analyses showed co-expression of the two markers in 98.2% of the cells (Figure 1B).

Microscopic evaluation of these cells (named as RP-MOC1) revealed polygonal shaped cells with a cobblestone morphology, characteristic of OSCC (Figure 1C). Species specific PCR evaluation confirmed the murine origin of the cells with no mammalian interspecies contamination (Table S1). Short tandem repeat (STR) profiling of RP-MOC1 cells confirmed a genetic profile identical to C57Bl/6 mouse strain consistent with the origin of the cells (Table S2). The growth curve of RP-MOC1 cells over a five-day period showed a typical “S” shape with a calculated doubling time of 37.6 ± 2.4 h (Figure 1D).

The in vitro behavior of RP-MOC1 cells was studied using wound healing, migration, and invasion assays. The wound healing assay showed the ability of RP-MOC1 cells to migrate with a near complete wound closure seen by 48 h (Figure 1E). Quantitative analyses using the transwell migration assay showed ~7-fold increase (*p* = 0.006) in the motility of these cells from 11.42 ± 1.46 cells (at 12 h) to 75.67 ± 11.68 cells (at 24 h) (Figure 1F). A three-fold increase (*p* = 0.049) in the number of cells invading through the matrigel coated transwell chamber by 24 h (189.9 ± 44.19 cells) compared to 12 h (60.21 ± 14.82 cells) (Figure 1G).

### 2.2. RP-MOC1 Cells Harbor Genetic Alterations Similar to Human Head and Neck Cancer

We performed exome sequencing at a depth of 20× and 30×, with 88% and 74% of targeted regions covered, respectively. The data were mapped to mm10 genome (reference strain C57BL/6NCr) and GENCODE GRCm38.75 annotation (gene set). Following filtration and alignment with GATK 3.5 against the reference genome, we found that the coding regions of RP-MOC1 genomes contained of 3404 single nucleotide variations (SNVs) and 146 small insertions and deletions (INDEL) (Supplementary File 1). Out of 3404 SNVs, 1727 (50%) missense variants were observed. The majority of the SNP variants found were G>T transversion (30.8%) followed by C>A transversion (28.6%). We compared the filtered SNVs against the mutation landscape of human head and neck SCC (http://www.cbioportal.org/public-portal/). The results showed that mutations of TP53, NFE2L2, CSMD3, STEAP4, UNC13C and NOTCH2 were found in the Single Nucleotide Polymorphism database (dbSNP) (Table S3). There were also 33 frameshift variants (22.6%) observed from the 146 INDELs (Supplementary File 1). Out of 1727 SNPs, 32 genes were found to associated with epithelial mesenchymal transition (Supplementary File 2).

### 2.3. Radiation Response of RP-MOC1 Cells in 2D Culture and as 3D Organoids

**Figure 2:**
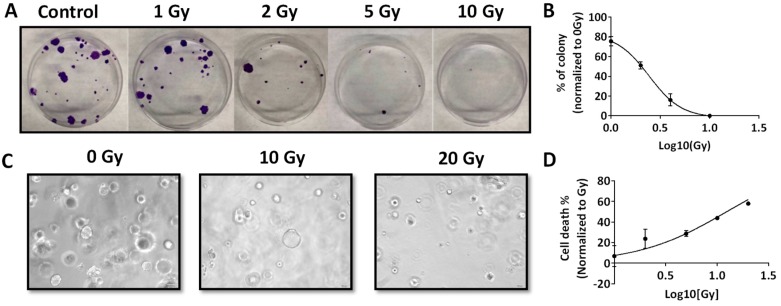
Radiation response of RP-MOC1 in 2D culture and 3D organoids. (**A**) Digital photographs of crystal violet stained RP-MOC1 cells on day 15 post radiation compared control. Radiation dose response curves of RP-MOC1 cells in 2D (**B**) RP-MOC1 organoids (**C**). Bright field microscopy images (**D**) of RP-MOC1 organoids showing a reduction in size post radiation. Data are expressed as mean ± SEM. Image was acquired at 20× magnification.

Next, we examined the response of RP-MOC1 cells to radiation in vitro. We exposed RP-MOC1 cells in 2D culture to increasing doses of radiation (0–10 Gy) and evaluated the response using the colony forming assay. At fifteen days post radiation, a 20%–100% reduction in colony formation was observed with increasing radiation dose from 1 to 10 Gy compared to controls (Figure 2A). The radiation dose to achieve 50% of cell death was 2.4 ± 0.48 Gy (Figure 2B). We also examined the response RP-MOC1 organoids to radiation (dose range 1–20 Gy). The morphology of the organoids was evaluated at 7 days post radiation treatment. Size (diameter measured in pixels) of the organoids was measured from bright field microscopic images (Figure 2C). The radiation response curves of RP-MOC1 organoid culture showed the lethal dose (LD_50_) mean value of 12.61 ± 1.22 Gy (Figure 2D). Compared to control (102.3 ± 6.37), 20 Gy radiation resulted in a significant reduction in size (52.88 ± 7.25) reflective of growth inhibition (*p* = 0.004). The difference in diameter between control and 10 Gy radiation (70 ± 15.18) was not statistically significant (*p* = 0.05).

### 2.4. Histopathologic Credentialing of RP-MOC1 Tumors

We performed histopathologic evaluation of the initial subcutaneous tumor and subsequent orthotopic allografts. In addition to hematoxylin and eosin (H&E) staining, tumor sections were immunostained for cytokeratin (Pan-CK), the proliferation marker, Ki-67, the epithelial marker, E-cadherin, and the mesenchymal marker, vimentin.

**Figure 3:**
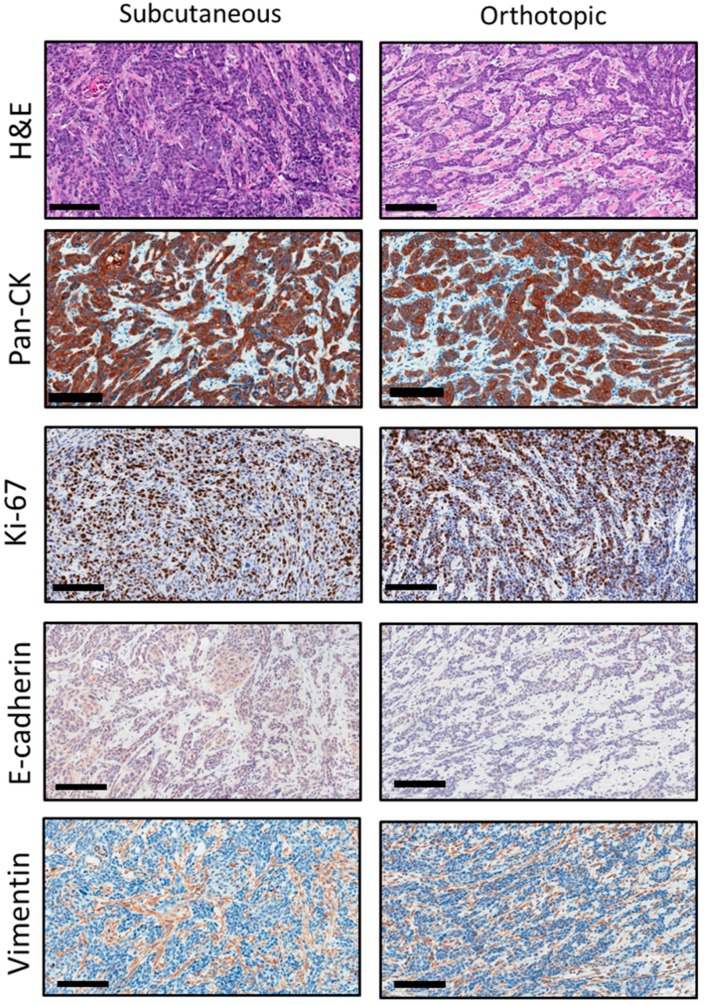
Histopathologic credentialing of RP-MOC1 tumors. Photomicrographs of histology and immunohistochemical staining of tumor sections from the initial subcutaneous tumor and the subsequent orthotopic tumor are shown. Hematoxylin and eosin (H&E) stained sections showed invasive keratinizing moderately differentiated squamous cell carcinoma. Image was acquired at 20× magnification. Scale bar: 100 μm. Immunostaining for cytokeratin (Pan-CK) and Ki-67 showed positive staining on the membrane and nuclear staining of the epithelial cells. Immunostaining for E-cadherin and vimentin showed negative staining on the membrane and cytoplasm stain of the epithelial cells. Images were acquired at 20× magnification. Scale bar: 100 μm.

Representative photomicrographs of H&E and immunostained sections of the initial subcutaneous tumor and subsequent orthotopic RP-MOC1 tumors are shown in Figure 3. Hematoxylin and eosin (H&E) stained sections showed invasive keratinizing moderately differentiated squamous cell carcinoma. The tumor cells showed vesicular to hyperchromatic nuclei with abundant eosinophilic cytoplasm and indistinct cellular outlines. Quantification of Ki-67 staining based on H-score did not reveal a significant difference (*p* > 0.05) in the proliferation index between subcutaneous (223 ± 56) and orthotopic (301 ± 49) tumors. The connective tissues expressed positive staining of vimentin compared to epithelial SCC cells in the initial subcutaneous tumor and orthotopic tumor grafts.

### 2.5. Radiation Response of Orthotopic RP-MOC1 Tumors in Immunocompetent and Immunodeficient Hosts

Building on the in vitro observations, we examined the response of orthotopic RP-MOC1 tumors to radiation in vivo. Non-invasive magnetic resonance imaging (MRI) was performed once every 3–4 days post implantation to assess tumor growth and response to radiation.

**Figure 4:**
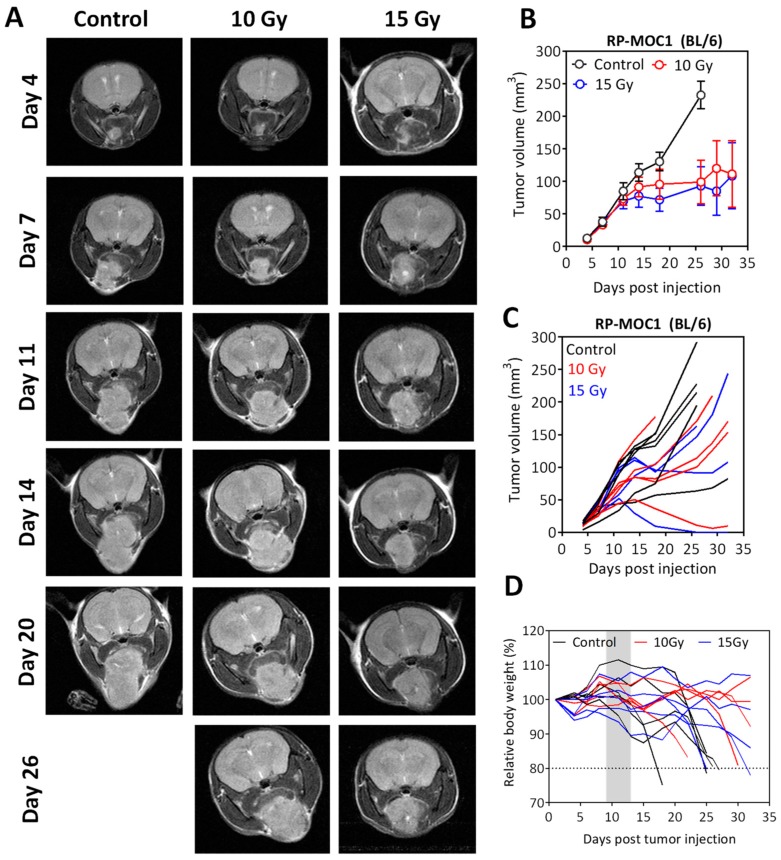
Radiation response of orthotopic RP-MOC1 tumors in immunocompetent mice. (**A**) Panel of images represents axial T2-weighted images of albino C57Bl/6 mice bearing orthotopic RP-MOC1 tumors during a 30-day monitoring period. Longitudinal MRI was performed once every 3–4 days during this period to assess tumor growth and response to radiation. Individual (**B**) and grouped (**C**) tumor growth curves based on MR-based tumor volume measurements obtained for animals in the control and radiation groups are shown. (**D**) Body weight measurements of animals in all three groups did not reveal any evidence of radiation toxicity. A gradual reduction in body weight associated with tumor growth was seen during the observation period.

Tumor-bearing albino C57Bl/6 mice were assigned to control or radiation groups ~10 days post tumor inoculation. Animals in the radiation arm received 10 Gy (5 daily fractions of 2 Gy) or 15 Gy (5 daily fractions of 3 Gy). The panel of images shown in Figure 4A represent axial T2-weighted images tumor bearing mice over a 30-day period. Tumor volumes were calculated from multi-slice T2-weighted images (Figure 4B,C). As evident from the images and the quantitative assessment, significant inhibition of tumor growth was seen with radiation at both doses compared to untreated controls. No significant difference in tumor volume was observed between 10 Gy and 15 Gy radiation exposure. Individual tumor growth curves illustrate the heterogeneity in response to radiation (Figure 4C). A gradual reduction in body weight associated with tumor growth was seen during the study period (Figure 4D).

**Figure 5:**
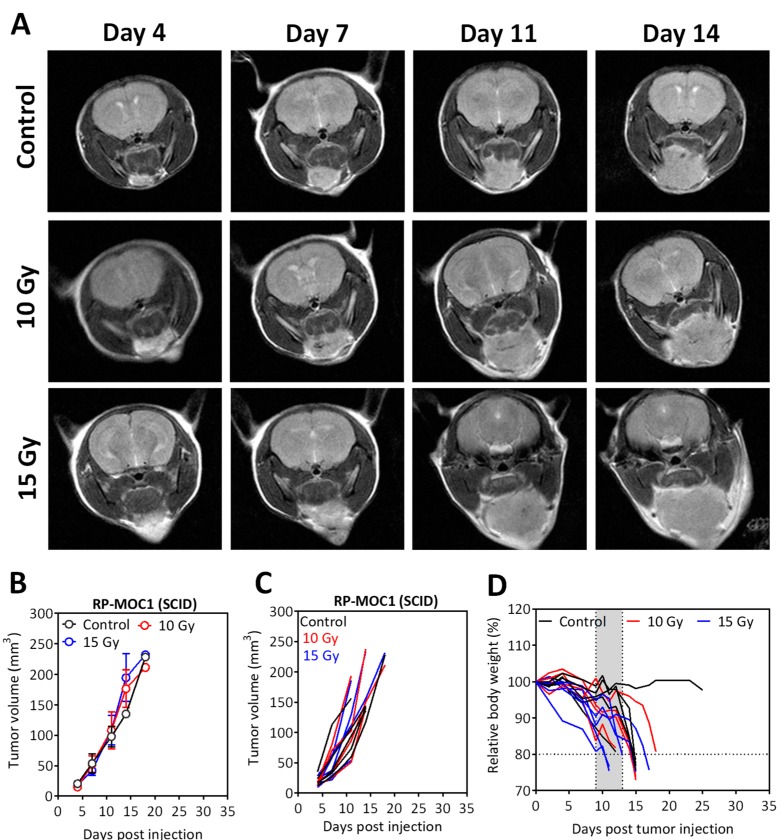
Radiation response of orthotopic RP-MOC1 tumors in SCID mice. (**A**) Panel of images represent axial T2-weighted images of SCID mice bearing orthotopic RP-MOC1 tumors over a 2-week monitoring period. Individual (**B**) and grouped (**C**) tumor growth curves based on MR-based tumor volume measurements obtained for animals in the control and radiation groups are shown. (**D**) Body weight measurements of animals in all three groups.

Comparative evaluation of the radiation response of RP-MOC1 tumors in immunodeficient mice (Figure 5) revealed no evidence of tumor growth inhibition following radiation treatment at both doses. The panel of images shown in Figure 5A represent T2-weighted images of SCID mice bearing orthotopic RP-MOC1 tumors over a two-week monitoring period. Grouped (Figure 5B) and individual (Figure 5C) MR-based tumor volume measurements for animals in the control and radiation groups are also shown. No difference in tumor volume was observed between control and radiated animals, highlighting the role of the immune system in tumor response to radiation.

## 3. Discussion

The translation of basic research into improved therapies for head and neck cancer patients will require the development of clinically relevant animal models that can capture the complex interactions between the tumor and the host. In the present study, we report on the establishment, cellular, genetic, and histopathologic credentialing of a new immunocompetent mouse model of OSCC. Our results illustrate the potential translational utility of this novel model as a valuable platform for the conduct of imaging-guided preclinical radiation trials against OSCC.

To generate the OSCC cell line, donor tongue tissue from a 4NQO exposed mouse was stained for the cancer stem cell marker, CD44 and the membrane glycoprotein, EpCAM and subsequently sorted using flow cytometry. These markers are highly expressed in OSCC and have been associated with an invasive and proliferative phenotype [28,29]. Mutational profiling of the cell line revealed a characteristic tobacco-associated signature (C:G > A:T transversions) that has been reported in smokers [30,31]. Exome sequencing revealed that RP-MOC1 cells harbor common somatic mutations of TP53, NFE2L2, CSMD3, STEAP4, UNC13C and NOTCH2 similar to human head and neck cancer. In addition to mutation of the bonafide tumor suppressor, TP53, we identified SNP missense mutation in NFE2L2, the loss of which reflects dysregulated oxidative stress [32,33]. STEAP4 is a metalloreductase known for its role in cell proliferation and metabolism that has been shown to be overexpressed in OSCC [34]. While UNC13C and STEAP4 mutations have been found human head and neck cancers, their role in oral carcinogenesis remains unclear. Similarly, deletion of CSMD1 gene has been associated with lymph node metastasis and poor prognosis in several cancers and is seen commonly in smokers [35]. We detected the less common NOTCH2 mutation in RP-MOC1 cells. Hijoka et al. have previously shown NOTCH2 overexpression in OSCC compared to normal oral mucosa, highlighting its potential role in oral tumorigenesis [36]. GSEA pathway analysis of SNP variant genes revealed association with epithelial-to-mesenchymal transition (EMT) consistent with the proliferative, migratory and invasive phenotype of RP-MOC1 cells in vitro.

Radiation therapy remains an integral part of standard of care for head and neck cancer patients [4,37]. We therefore examined the radiation response of RP-MOC1 cells in 2D and 3D organoid cultures. The LD50 for RP-MOC1 cells in 2D culture was 2 Gy (single dose) while 3D organoid cultures exhibited a higher LD50. Our observations are consistent with a previous study by Storch et al. [38] in which 3D cultures of head and neck and lung cancers exhibited reduced DNA double strand breaks and increased survival as a result of cell–cell and cell–matrix interactions [39]. Building on the in vitro observations, we investigated the histology, growth kinetics and response of our model in vivo. Consistent with the proliferative and invasive phenotype observed in vitro, RP-MOC1 tumors grew orthotopically in the floor of the mouth and demonstrated invasive squamous histology with positive immunostaining for pan-cytokeratin and Ki67. In vivo radiation response studies performed in orthotopic RP-MOC1 tumors established in immunocompetent C57Bl/6 showed significant tumor growth inhibition on MRI examination following 10 Gy and 15 Gy fractionated radiation regimens. Given the well-recognized immune-mediated effects of radiation, we examined the radiation response of RP-MOC1 tumors in immunodeficient mice. Non-invasive MRI showed no evidence of antitumor response with the two regimens in SCID mice. This observation is consistent with a previous work by Liang et al. on the role of host immune responses in tumor response to radiation [40]. In the study, Liang and colleagues demonstrated prolonged stable responses or partial responses as a result of both tumor cell proliferation and immune cell infiltration resulting in ‘radiation-induced tumor equilibrium’. Consistent with our observations, the study demonstrated a complete lack of radiotherapeutic efficacy in SCID mice compared to immunocompetent BALB/c mice.

Our observations should be interpreted in the context of the limitations of our study. While preclinical models are essential tools for cancer research, no experimental tumor model can fully recapitulate the disease characteristics seen in OSCC patients. Syngeneic models enable conduct of studies in immunocompetent hosts. However, the murine origin of the tumors needs to be considered. The observed radiation response in our model system may not directly reflect the sensitivity of human OSCC to radiation. In this regard, we have previously examined the radiation response of patient derived xenograft (PDX) models of OSCC [9]. However, human tumor xenograft models necessitate the use of immunodeficient hosts. While our orthotopic tumor model recapitulates the aggressive growth characteristics of the disease, the stromal and vascular components in syngeneic models and human tumor xenograft models are of murine origin. Indeed, no animal model can fully recapitulate human tumors in terms of their biology or therapeutic response. Oral cancers represent a heterogeneous group of neoplasms with varying biological behavior. In our study, we successfully established and credentialed the response of one sub type of these cancers, namely, tongue cancer. We therefore submit that preclinical investigation into strategies aimed at sensitizing tumors to radiation should include systematic evaluation in syngeneic murine models and human OSCC xenograft models that account for the biological heterogeneity of this patient population. In this regard, our 3D organoid platform could be used as a first step for in vitro screening of potential ‘radiosensitizers’ prior to in vivo evaluation. Our in vivo orthotopic model could also serve as a valuable tool to examine the activity of novel immunotherapeutic strategies against this disease. While immune checkpoint inhibitors have recently received approval for clinical use in head and neck cancer patients [41], the optimal dose, schedule and sequence of combining checkpoint inhibitors with standard of care chemoradiation regimens is still unclear [42,43]. As such, several ongoing trials are investigating the combination of these immune-oncology agents with chemotherapy, radiation and targeted therapies [43]. Given the costs associated with large scale randomized trials, our model could be useful in the conduct of imaging-guided preclinical trials of these immunomodulatory agents with radiation. The model could also be used to understand mechanisms of resistance to radiation and immune checkpoint inhibitors. To address some of these questions, we have begun developing additional murine OSCC lines and will report on our findings in the future.

## 4. Materials and Methods

### 4.1. Establishment of the RP-MOC1 Cell Line and Culture Conditions

To induce oral lesions, female C57BL/6NCr mice (Charles River, Wilmington, MA, USA) were exposed to the carcinogen 4NQO (Sigma-Aldrich, St. Louis, MO, USA) in drinking water for 16 weeks. An exophytic tongue lesion from a donor mouse was transplanted subcutaneously into a naïve recipient animal. When the resultant tumor reached a volume of ~200 mm^3^, the tissue was excised, enzymatically dissociated and digested in DMEM 1× media containing 1% of penicillin-streptomycin (Life Technologies, Carlsbad, CA, USA), and 5 mg/mL of collagenase type IV (Life Technologies) for 3 h. After digestion, the cells were trypsinized and washed with cell culture media consisting of DMEM 1× with 10% fetal bovine serum and 1% penicillin-streptomycin (Life Technologies). Finally, the cells were filtered through a 40 μm cell strainer and the cell suspension was seeded in culture flask with cell culture media at 37 °C, 5% CO_2_ for 7 days prior to cell sorting using flow cytometry.

### 4.2. Fluorescence Activated Cell Sorting (FACS)

One million cells were labeled with a cocktail of antibodies containing CD44 BV421 (clone IM7, BD Biosciences, San Jose, CA, USA) and CD326 PE (clone G8.8, BD Biosciences, San Jose, CA, USA) for 30 min at 4 °C. Isotype BV421 (Rat IgG2b, κ; BD Biosciences) and isotype PE (IgG2a, κ; BD Biosciences, San Jose, CA, USA) were used as controls to determine the separation between positive and negative cells. Compensation was performed using single cells labeled with appropriate CD44 BV421 (clone IM7, BD Biosciences) and CD326 PE (clone G8.8, BD Biosciences, San Jose, CA, USA), respectively. Epithelial cells, defined by their positive epithelial cell adhesion molecule (CD326; EpCAM) and CD44 expression were sorted (FACS ARIA, BD Biosciences, San Jose, CA, USA) and a minimum of 500,000 cells were collected. Sorted cells were cultured immediately in cell culture media at 37 °C, 5% CO_2_. Flow data were analyzed using FCS Express 6.0 (De Novo Software, Pasadena, CA, USA).

### 4.3. Authentication and IMPACT Testing of RP-MOC1

To verify the species origin and mycoplasma contamination of RP-MOC1, the cells were sent to IDEXX Laboratories, Minnesota, USA for STR DNA fingerprinting and PCR species evaluation performed using the CellCheckTM Mouse kit.

### 4.4. Exome Sequencing

Genomic DNA was prepared from RP-MOC1 cells in culture (Qiagen DNeasy Blood & Tissue Kit, Hilden, Germany) and subjected into Illumina libraries according to the manufacturer’s protocol (Illumina Inc, San Diego, CA, USA). Mutation reads were normalized to the reference C57BL/6NCr genome (mm10 genome) and a genomic database (GENCODE GRCm38.75) of 18 commonly used strains of inbred laboratory mice. Mutational data on human OSCC was obtained from publicly available data in TCGA; cBioPortal (http://www.cbioportal.org/public-portal/). The identified SNPs were subjected to Gene Set Enrichment Analysis (GSEA) pathway analysis [44].

### 4.5. MTT Assay

Cells (4.0 × 10^3^ cells) were suspended in 500 μL of cell culture media, plated in 24 well microplates and cultured for 24, 48, 72, 96 and 120 h. Fifty microliters (μL) of 5 mg/mL MTT (Calbiochem, San Diego, CA, USA) was added and after 4 h of incubation, 500 μL of each DMSO (Sigma-Aldrich, MO, USA) was added. The absorbance was measured at 570 nm (A570) and reference absorbance was 670 nm (A670) using Synergy HTX Multi-Mode Reader (BioTek, Winooski, VT, USA). The cell viability is proportional to A570-A670. Two independent experiments were performed with nine replicates.

### 4.6. Wound Healing Assay

Wound healing migration assay was performed using a 35-mm μ-Dish (ibidi GmbH, Munich, Germany). A total of 70 μL of 35,000 cells were seeded into each chamber of the cell culture insert for overnight. The next day, cells were treated with 5 µg/mL of Mitomycin-C (Calbiochem, San Diego, CA, USA) for 2 h at 37 °C before the cell culture insert was gently removed with sterile forceps. The cultures were washed twice with PBS. Then, 2 mL of cell culture media was added into the 35 mm μ-Dish. The cells that migrated into the denuded area were captured with microscope at 4× magnification. Two independent experiments were performed for this assay.

### 4.7. Transwell Migration Assay

Cells were tested in transwell migration assay using cell culture inserts with PET membrane of 8 µm pore sizes, according to the manufacturer’s protocol (Corning, Tewksbury, MA, USA). Briefly, cells were cultured and serum starved for 24 h. Cells were then harvested and suspended in serum free cell culture media at a concentration of 3.0 × 10^5^ cells/mL. In the 24 well plate, inserts were placed into wells containing 500 μL of cell culture media that acted as a chemoattractant, and followed by 200 μL of serum starved cells were seeded onto the insert for 12 and 24 h, respectively. Post 12- and 24-h incubation, the non-migrated cells were first eliminated by scraping with wet cotton swabs, while the bottom of the membrane was fixed and stained with 0.1% crystal violet (Sigma-Aldrich, St. Louis, MO, USA) in 20% methanol for 2 h at room temperature. Membranes were washed with water to remove excessive stained and air dried. The membrane was then viewed and captured under the microscope at 20× magnification. The number of stained cells were counted in 4 randomly chosen microscopic fields and averaged. Three independent experiments were performed with 2 replicates.

### 4.8. Matrigel Invasion Assay

Experiments were carried out as described in the transwell migration assay except that the inserts used were pre-coated with Matrigel basement matrix from Biocoat Matrigel 24 well invasion chamber (Corning, Tewksbury, MA, USA). Briefly, cells were cultured and serum starved for 24 h. Cells were then harvested and suspended in serum free cell culture media at a concentration of 5.0 × 10^5^ cells/mL. In the 24 well plate, inserts were rehydrated with serum free cell culture media for 2 h before they were placed into wells containing 750 µL of cell culture media that acted as chemoattractant. This was followed by seeding 500 µL of serum starved cells onto the insert for 12 and 24 h. Post 12- and 24-h incubation, non-migrated cells were scraped by wet cotton swab, while the bottom of the membrane was fixed and stained with 0.1% crystal violet (Sigma-Aldrich, St. Louis, MO, USA) in 20% methanol for 2 h at room temperature. Membranes were washed with water, air dried and microscopic images captured at 20× magnification. The number of stained cells were counted in 4 randomly chosen microscopic fields and averaged. Three independent experiments were performed with 2 replicates.

### 4.9. Clonogenic Assay

Five hundred thousand cells were plated to five different T-25 flasks. Next day, the T-25 flasks were radiated by the irradiator (Shepherd Mark I Model 68, 4000 Ci Cesium 137 source irradiator, J.L. Shepherd and Associates), with 1, 2, 4, 10 Gy at position 2. Post 24 h, 120 cells were plate to 60 mm petri dish. Cell culture media was refreshed every 3 days. After 15 days of culture, the colonies were fixed and stained with 0.1% crystal violet (Sigma-Aldrich, St. Louis, MO, USA) in 20% methanol for 2 h at room temperature. Colonies were counted manually. Three independent experiments were performed in triplicate.

### 4.10. Establishment and Radiation Response Assessment of RP-MOC1 Organoids

To establish RP-MOC1 organoids, 40 μL of matrigel (Corning, Tewksbury, MA, USA) was used to suspend single RP-MOC1 cells that were plated in 6-well plates in organoid media. In order to prepare organoid media, Advanced DMEM/F12 (Life Technologies, Carlsbad CA, USA) is supplemented with 1× GlutaMAX (Life Technologies, Carlsbad, CA, USA), 10 mM HEPES (Life Technologies, Carlsbad, CA, USA) and 100 μg/mL Primocin (InvivoGen, San Diego, CA, USA), 50% of L-WRN (CRL3276) conditioned media containing signaling factors Wnt3a, R-spondin-3, and Noggin (ATCC, Manassas, VA, USA), 1× B27 supplement (Life Technologies, Carlsbad, CA, USA), 1× N2 supplement (Life Technologies, Carlsbad, CA, USA), 10 mM Nicotinamide (Sigma-Aldrich, St. Louis, MO, USA), 1.25 mM *N*-Acetyl-l-cysteine (Sigma-Aldrich, St. Louis, MO, USA), 4 µM SB 202190 (Peprotech, Rocky Hill, NJ, USA), 10 ng/mL Human FGF10 (Peprotech, Rocky Hill, NJ, USA), 100 ng/mL Human EGF (Peprotech, Rocky Hill, NJ, USA) and 500 nM A 83-01 (Peprotech, Rocky Hill, NJ, USA).RP-MOC1 organoids were cultured in humidified cell culture incubators at 37 °C in 5% CO2. To assess the radiation response of RP-MOC1 organoid, 20 μL of matrigel (Corning, Tewskbury, NY, USA) that suspended with 5000 RP-MOC1 cells were seeded in a white 96 well plate. Post 24 h, cells were radiated by the irradiator (Shepherd Mark I Model 68, 4000 Ci Cesium 137 source irradiator, J.L. Shepherd and Associates), with 1, 2, 5, 10 and 20 Gy at position 2. After 7 days, CellTiter-Glo^®^ 3D cell viability assay (Promega, Madison, WI, USA) was used to measure the number of viable cells based on quantitation of the ATP present, which signals the presence of metabolically active cells using luminescent detector (Synergy HTX, BioTek, Winooski, VT, USA). The difference in luminescent signal post radiation normalized to control (0 Gy) was used as the measurement of cell viability. Cell death was calculated as the difference in viable cells from 100% viable control. The radiation dose that able to achieve 50% cell death (EC50) was determined from absorbance signal versus concentration curve using GraphPad software by applying the nonlinear regression and the equation log (agonist) vs. normalized response. All experiments were carried out in triplicate and performed in three independent experiments. Bright field microscopy images of the organoid were captured and the diameter of the control (0 Gy), 10 Gy and 20 Gy were measured using cellSens Standard 1.6 software (Olympus, Center Valley, PA, USA).

### 4.11. In Vivo Radiation Studies

Experimental studies were carried out using eight-to-twelve week old female C.B 17 severe combined immunodeficient (SCID) (C.B-Igh-1^b^/IcrTac-Prkd^scid^; Laboratory Animal Shared Resource, Roswell Park) and C57BL/6NCr mice with an average body weight ~20 g. Mice were kept in sterile micro-isolator cages (4–5 mice per cage) in a pathogen-free environment and provided with standard chow/water and maintained on 12 h light/dark cycles in a HEPA-filtered environment. Animals were injected with 1 × 10^6^ RP-MOC1 cells orthotopically in the floor of mouth. Seven to ten days post injection, non-invasive magnetic resonance imaging (MRI) was performed to confirm tumor growth. Tumor-bearing mice were randomized into control (*n* = 5, untreated) or one of two radiation arms (10 Gy; 5 doses of 2 Gy radiation delivered on consecutive days (*n* = 5); 15 Gy; 5 doses of 3 Gy radiation delivered on consecutive days (*n* = 5). Irradiation was performed using the Philips RT 250 Orthovoltage X-ray unit (Philips Medical Systems, Andover, MA, USA). Radiation was delivered through an axial beam directed to the tumor in the floor of the mouth. A lead shield was utilized to protect the thoracic cavity from exposure to radiation. Body weights of the animals were measured once every 3 days throughout the duration of the study as a measure of toxicity. Experimental procedures were performed under aseptic conditions and in accordance with protocols approved by the Institutional Animal Care and Use Committee (Protocol #1183M; Original approval: July 2017; Renewal; September 2019; Animal welfare assurance number: A-3143-01.

### 4.12. Magnetic Resonance Imaging

Longitudinal MRI examinations were performed using a 4.7T/33-cm horizontal bore magnet (GE NMR Instruments, Fremont, CA, USA) incorporating AVANCE digital electronics (Bruker Biospec with Paravision 3.0.2; Bruker Medical Inc., Billerica, MA, USA). Animals were anesthetized using 2.5% Isoflurane (Benson Medical Industries, Markham, ON, Canada) prior to and during imaging. Tumor volumes were calculated from multi-slice, axial T2-weighted spin echo images incorporating RARE (rapid acquisition with relaxation enhancement) [45].

### 4.13. Histology and Immunohistochemistry

Mice were humanely euthanized according to recommendations of the Panel on Euthanasia of the American Veterinary Medical Association and tumor tissues resected for further processing. Tumor tissues were fixed in 10% neutral buffered formalin (Sigma-Aldrich, St. Louis, MA, USA) and embedded in paraffin. Tissues were sectioned at a thickness of 4 μm, mounted on positively charged slides, and stained for hematoxylin and eosin (H&E) stain and immunohistochemistry. Immunohistochemistry was performed on FFPE sections of the tissues using the Envision technique, Dako Real EnVision Detection System and Peroxidase/DAB+ (Dako Corporation, Carpinteria, CA, USA) according to the manufacturer’s protocol (Table S4). Briefly, FFPE sections were deparaffinized in xylene and rehydrated in ethanol series. Antigen retrieval was carried out using pre-heat steamer for 30 min. The sections were immersed in blocking solution (Dako Corporation, Carpinteria, CA, USA) for 10 min at room temperature followed for blocking the endogenous peroxidase activity. The sections were then incubated with serum free blocker (Dako Corporation, Carpinteria, CA, USA) for an hour at room temperature followed by 30 min to an hour incubation of primary antibody at room temperature (Pan cytokeratin, Abcam, Cambridge, UK, Catalog #ab9377; E-cadherin, Cell Signaling Technology, Danvers, MA, USA, Catalog #3195; Vimentin, Boster Biological Technology, Pleasanton, CA, USA, Catalog # PB9359) Ki-67, R&D Systems Inc., Minneapolis, MN, USA Catalog #MAB7617). After washing with TBS (pH 7.4) plus 0.1% Tween 20 (Bio-Rad Laboratories, Hercules, CA, USA), sections were incubated with the peroxidase labeled secondary antibody from the Envision kit for an hour for the immunoreactivity performances. Finally, sections were stained with 3′3 diaminobenzidine substrate chromogen, (Dako Corporation, Carpinteria, CA, USA) counterstained with Harris hematoxylin, dehydrated and mounted. Images were captured at 20× magnification. Ki-67 stained specimens were captured at 40× magnification and eight random areas were analyzed using NIH Image J software (NIH, Bethesda, MD, USA). Intensity of the stain was classified into 0 = negative/no stain, 1 = weak, 2 = moderate and 3 = strong. For each image, an observer who was blinded to the identity of the samples assessed 200 cell nuclei for staining intensity and assigned a value from 0 to 3. The H-scores for Ki-67 were calculated based on the sum of scores from each sample between subcutaneous and orthotopic tumor samples.

### 4.14. Sample Sizes and Statistics

All statistical analysis was performed using GraphPad version 7.00 for Windows (GraphPad Software, San Diego, CA, USA). The RP-MOC1 in vitro doubling time was determined by least-squares fitting of single-exponential curves to the cell viability against time (day). The comparison between the 12-h and 24-h migrated and invaded cells from transwell migration and invasion assays were compared using unpaired Student’s *t*-test. H-scores from Ki-67 immunostained sections of subcutaneous and orthotopic tumors were also analyzed using unpaired Student’s *t*-test. Non-invasive imaging was performed using 4–5 animals per cohort. Comparisons of organoid for 0 Gy, 10 Gy and 20 Gy and animal tumor volume across cohorts were analyzed using one-way ANOVA test. All results are expressed as mean values ± SEM of at least three independent experiments, except when otherwise indicated. *p*-values of <0.05 were considered statistically significant.

## 5. Conclusions

We have successfully established and credentialed a novel immunocompetent model of OSCC that exhibits a mutational and histologic profile similar to human disease. The model can serve as a valuable platform for evaluation of combination strategies to enhance radiotherapeutic efficacy against this deadly disease.

## Supplementary Materials

The following are available online at https://www.mdpi.com/2072-6694/12/3/579/s1, Table S1: Species-specific PCR evaluation/Interspecies contamination evaluation of RP-MOC1, Table S2: Genetic profile of RP-MOC1, Table S3: Somatic mutations in the RP-MOC1 cell line, Table S4: List of antibodies that use for immunohistochemistry, Supplementary File 1: Summary of SNP and INDEL analyses, Supplementary File 2: Summary of gene list and GSEA datatitle.
