# Supplementary material for: Development and Radiation Response Assessment in A Novel Syngeneic Mouse Model of Tongue Cancer: 2D Culture, 3D Organoids and Orthotopic Allografts
Source: Cancers (Basel). 2020 Mar 2;12(3):579. doi: 10.3390/cancers12030579 (PMC7139805; doi:10.3390/cancers12030579)
Supplement: Supplementary file 1 [file cancers-12-00579-s001.zip › cancers-699887-supplementary - final/cancers-699887 - supplementary - proof.pdf]

Supplementary Materials

Development and Radiation Response Assessment in A Novel Syngeneic Mouse Model of Tongue Cancer: 2D Culture, 3D Organoids and Orthotopic Allografts

Vui King Vincent-Chong and Mukund Seshadri

Table S1. Species-specific PCR evaluation/Interspecies contamination evaluation of RP-MOC1. The cells were confirmed to be of murine origin and no mammalian interspecies contamination was detected.

Species	Result
Mouse	Positive
Rat	Negative
Human	Negative
Chinese hamster	Negative
African green monkey	Negative

Table S2. Genetic profile of RP-MOC1. The genetic profile of RP-MOC1 was evaluated using a panel of STR markers for genotyping. Genetic testing of the sample confirmed the C57Bl/6 origin of the cells with no cross-contamination with any non-C57Bl/6 mouse strain origin cell lines.

Marker Name	Sample Results	C57BL/6NCr
MCA-4-2	20.3	20.3
MCA-5-5	17	17
MCA-6-4	19	17
MCA-6-7	15	15
MCA-9-2	18	18
MCA-12-1	17	17
MCA-15-3	22.3	22.3
MCA-18-3	16	16
MCA-X-1	27	27

Table S3. Somatic mutations in the RP-MOC1 cell line.

	Symbol	Type	mm10Symbol	Eff	Change
1	TP53	SNP	Trp53	missense_variant & splice_region_variant	p.Glu221Asp
2	TP53	SNP	Trp53	missense_variant & splice_region_variant	p.Ser258Asn
3	NFE2L2	SNP	Nfe2l2	missense_variant	p.Gly363Val
4	CSMD3	SNP	Csmd3	missense_variant	p.Thr2886Ile
5	STEAP4	SNP	Steap4	stop_gained	p.Trp286
6	UNC13C	SNP	Unc13c	missense_variant	p.Gln2115Lys
7	UNC13C	SNP	Unc13c	missense_variant	p.Ser134Ile
8	NOTCH2	SNP	NOTCH2	missense_variant	c.2379G>T

Table S4. List of antibodies that use for immunohistochemistry.

Marker	Antibody Clone	Dilution and Time	Antigen Retrieval	Source and Catalogue
Ki-67	1297A	1.125, 20 minutes, room temperature	10mM Tris EDTA (pH 9.0)	R&D Systems, Inc (MAB7617)
Pan Cytokeratin	AE1/AE3	1:200, 1 hour, room temperature	10mM Tris EDTA (pH 9.0)	Abcam (ab9377)
E-cadherin	24E10	1:1000, 1 hour, room temperature	10mM citrate buffer (pH 6.0)	Cell Signaling Technology (3195)
Vimentin		1.100, 30 minutes, room temperature	10mM Tris EDTA (pH 9.0)	Boster Biological Technology (PB9359)

© 2020 by the authors. Licensee MDPI, Basel, Switzerland. This article is an open access article distributed under the terms and conditions of the Creative Commons Attribution (CC BY) license (http://creativecommons.org/licenses/by/4.0/).
