# Supplementary material for: Development and Radiation Response Assessment in A Novel Syngeneic Mouse Model of Tongue Cancer: 2D Culture, 3D Organoids and Orthotopic Allografts
Source: Cancers (Basel). 2020 Mar 2;12(3):579. doi: 10.3390/cancers12030579 (PMC7139805; doi:10.3390/cancers12030579)
Supplement: Supplementary file 1 [file cancers-12-00579-s001.zip › cancers-699887-supplementary - final/SuppFiles1-2.pdf]

SnpEff: Variant analysis

Contents

Summary
Variant rate by chromosome
Variants by type
Number of variants by impact
Number of variants by functional class
Number of variants by effect
Quality histogram
InDel length histogram
Base variant table
Transition vs transversions (ts/tv)
Allele frequency
Allele Count
Codon change table
Amino acid change table
Chromosome variants plots
Details by gene

Summary

**Genome**	GRCm38.75
**Date**	2018-06-04 16:53
**SnpEff version**	SnpEff 4.3t (build 2017-11-24 10:18), by Pablo Cingolani
**Command line arguments**	SnpEff -stats ../output/SnpEff/RP-mSCC1-P30-_INDEL_snpEff_summary.html -no-intergenic -no-intron GRCm38.75 ../output/Variants_Filtered/RP-mSCC1-P30-_INDEL_PASS.vcf
**Warnings**	87
**Errors**	1
**Number of lines (input file)**	146
**Number of variants (before filter)**	146
**Number of not variants (i.e. reference equals alternative)**	0
**Number of variants processed (i.e. after filter and non-variants)**	146
**Number of known variants (i.e. non-empty ID)**	0 (0%)
**Number of multi-allelic VCF entries (i.e. more than two alleles)**	0
**Number of effects**	230
**Genome total length**	4,343,312,700
**Genome effective length**	2,633,776,673
**Variant rate**	1 variant every 18,039,566 bases

Variants rate details

Chromosome	Length	Variants	Variants rate
1	195,471,971	15	13,031,464
2	182,113,224	9	20,234,802
3	160,039,680	8	20,004,960
4	156,508,116	10	15,650,811
5	151,834,684	11	13,803,153
6	149,736,546	11	13,612,413
7	145,441,459	8	18,180,182
8	129,401,213	11	11,763,746
9	124,595,110	8	15,574,388
10	130,694,993	6	21,782,498
11	122,082,543	8	15,260,317
12	120,129,022	2	60,064,511
13	120,421,639	4	30,105,409
14	124,902,244	1	124,902,244
15	104,043,685	6	17,340,614
16	98,207,768	4	24,551,942
17	94,987,271	7	13,569,610
18	90,702,639	3	30,234,213
19	61,431,566	9	6,825,729
M	1	0	0
X	171,031,299	4	42,757,824
**Total**	**2,633,776,673**	**146**	**18,039,566**

Number variants by type

Type	Total
**SNP**	0
**Total**	**146**

Type	Total
MNP	0
INS	24
DEL	122
MIXED	0
INV	0
DUP	0
BND	0
INTERVAL	0
Total	146

Number of effects by impact

Type (alphabetical order)	Count	Percent
HIGH	38	16.594%
LOW	14	6.114%
MODERATE	2	0.873%
MODIFIER	175	76.419%

Number of effects by functional class

Type (alphabetical order)	Count	Percent

Missense / Silent ratio: 0

Number of effects by type and region

Type

Type (alphabetical order)	Count	Percent
3_prime_UTR_variant	9	3.488%
5_prime_UTR_variant	3	1.163%
disruptive_inframe_deletion	1	0.388%
disruptive_inframe_insertion	1	0.388%
downstream_gene_variant	28	10.853%
frameshift_variant	33	12.791%
intergenic_region	3	1.163%
intragenic_variant	4	1.55%
intron_variant	114	44.186%
non_coding_transcript_exon_variant	4	1.55%
splice_acceptor_variant	4	1.55%
splice_donor_variant	5	1.938%
splice_region_variant	20	7.752%
upstream_gene_variant	29	11.24%

Region

Type (alphabetical order)	Count	Percent
DOWNSTREAM	28	12.227%
EXON	38	16.594%
INTERGENIC	3	1.31%
INTRON	96	41.921%
SPLICE_SITE_ACCEPTOR	4	1.747%
SPLICE_SITE_DONOR	1	0.437%
SPLICE_SITE_REGION	14	6.114%
TRANSCRIPT	4	1.747%
UPSTREAM	29	12.664%
UTR_3_PRIME	9	3.93%
UTR_5_PRIME	3	1.31%

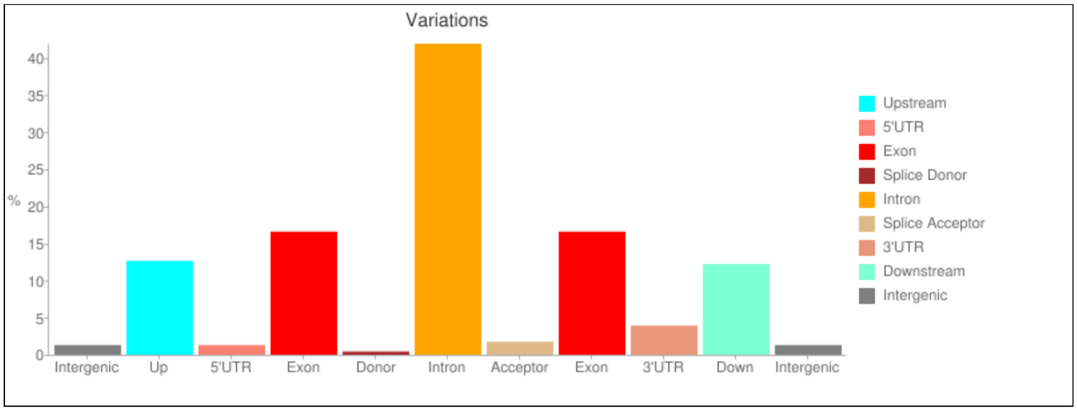

Quality:

Min	35
Max	16,107
Mean	890.233
Median	252.5
Standard deviation	1,877.942
Values	35,36,42,45,48,53,54,55,57,58,60,64,66,67,68,70,71,72,74,76,79,88,90,91,95,97,101,102,104,107,108,110,112,113,114,117,121,122,123,124,127,132,1
Count	1,1,1,1,1,1,1,2,1,1,2,3,1,1,1,1,1,3,1,1,1,1,1,1,1,1,1,1,1,1,1,1,1,2,1,1,1,1,1,1,1,1,1,2,1,1,2,1,2,2,1,1,1,1,1,2,1,1,1,1,1,1,1,1,2,1,1,1,1,1,1,1,1,1,1,1,1,1,1,

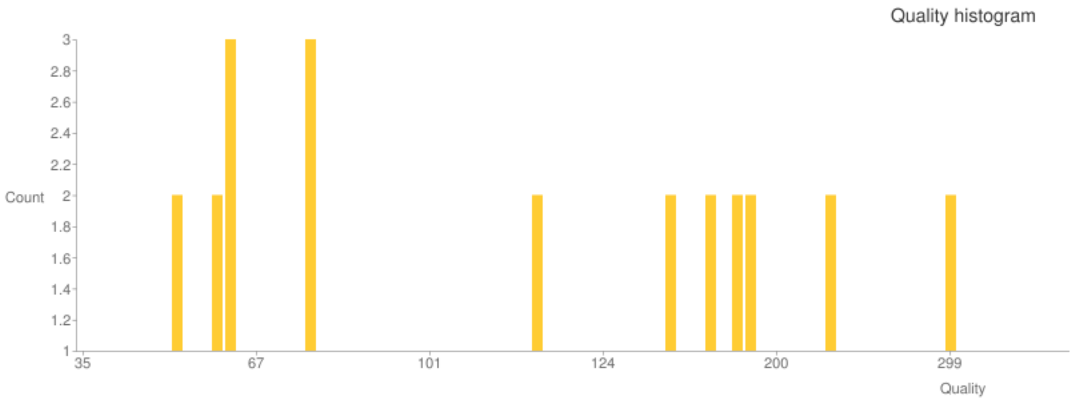

Insertions and deletions length:

**Min**	0
**Max**	14
**Mean**	1.021
**Median**	1
**Standard deviation**	1.412
**Values**	0,1,11,14
**Count**	20,124,1,1

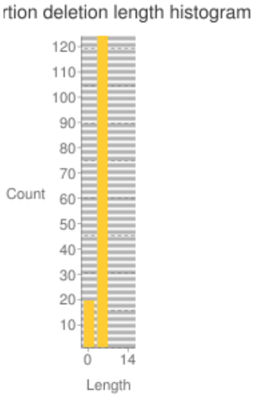

Base changes (SNPs)

	A	C	G	T
**A**	0	0	0	0
**C**	0	0	0	0
**G**	0	0	0	0
**T**	0	0	0	0

Ts/Tv (transitions / transversions)

Note: Only SNPs are used for this statistic.
Note: This Ts/Tv ratio is a 'raw' ratio (ratio of observed events).

**Transitions**	0
**Transversions**	0
**Ts/Tv ratio**	0

All variants:

`No results available (empty input?)`

Only known variants (i.e. the ones having a non-empty ID field):

`No results available (empty input?)`

Allele frequency

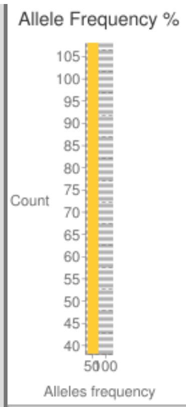

**Min**	50
**Max**	100
**Mean**	63.014
**Median**	50
**Standard deviation**	22.015
**Values**	50,100
**Count**	108,38

Allele Count

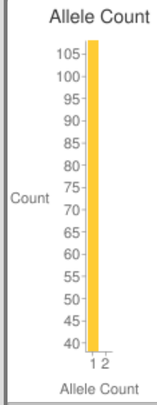

**Min**	1
**Max**	2
**Mean**	1.26
**Median**	1
**Standard deviation**	0.44
**Values**	1,2
**Count**	108,38

Hom/Het per sample

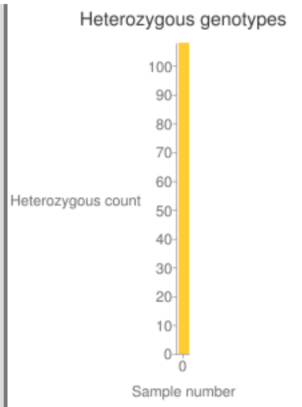

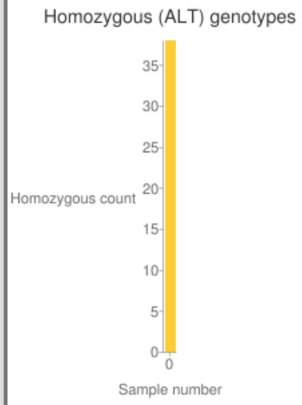

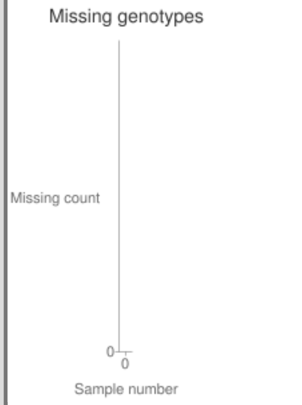


```
Sample_names , RS-03044064
Reference , 0
Het , 108
Hom , 38
Missing , 0
```

Codon changes

How to read this table:
- Rows are reference codons and columns are changed codons. E.g. Row 'AAA' column 'TAA' indicates how many 'AAA' codons have been replaced by 'TAA' codons.
- Red background colors indicate that more changes happened (heat-map).
- Diagonals are indicated using grey background color
- WARNING: This table may include different translation codon tables (e.g. mamalian DNA and mitochondrial DNA).

	-	ACC	ACG	AGA	ATA	ATG	CAA	CAC	CAG	CCA	CCC	CCG	CCT	CGG	CTG	CTT	GAC	GAG	GAT	GCC	GGA	GGC	GGT
-											1	1									1	1	1
ACC																	1						
ACG	1																						
AGA	1																						
ATA	1																						
ATG	1																						
CAA	2																						
CAC	1																						
CAG								1															
CCA																							
CCC	3									1		1											
CCG																							

	-	ACC	ACG	AGA	ATA	ATG	CAA	CAC	CAG	CCA	CCC	CCG	CCT	CGG	CTG	CTT	GAC	GAG	GAT	GCC	GGA	GGC	GGT
CCT																				1			
CGG	2																						
CTG	3																						
CTT	1																						
GAC																							
GAG	1																						
GAT	2																						
GCC	1																						
GGA																							
GGC	1																						
GGT	1								1														
GTA																							
GTG	2																						
GTT																							
TCC	1																						
TGC	2																						

Amino acid changes

How to read this table:
- Rows are reference amino acids and columns are changed amino acids. E.g. Row 'A' column 'E' indicates how many 'A' amino acids have been replaced by 'E' amino acids.
- Red background colors indicate that more changes happened (heat-map).
- Diagonals are indicated using grey background color
- WARNING: This table may include different translation codon tables (e.g. mamalian DNA and mitochondrial DNA).

	-	?	A	C	D	E	G	H	I	L	M	P	Q	R	S	T	V
-		7					3					2					
?																	
A	1																
C	2																
D	2																
E	1																
G	2												1				
H	1																
I	1																
L	4																
M	1																
P	3		1									2					
Q	2							1									
R	3																
S	1																
T	1				1												
V	2																3

Variants by chromosome

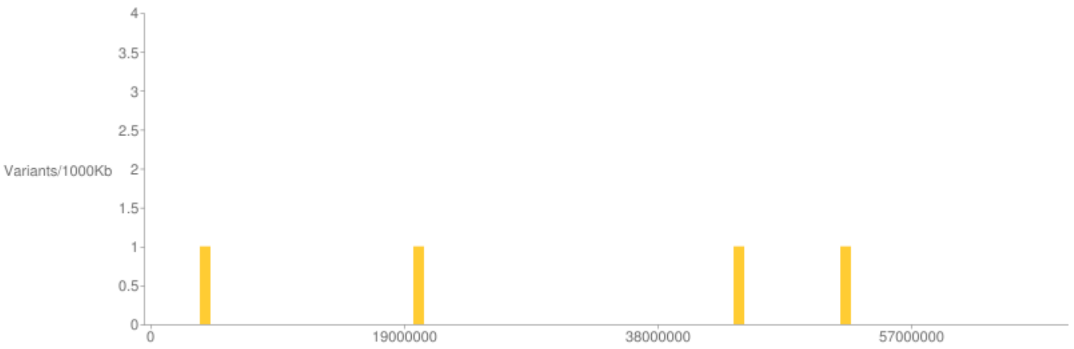

1, Position,0,1000000,2000000,3000000,4000000,5000000,6000000,7000000,8000000,9000000,10000000,11000000,12000000,130000
1,Count,0,0,0,0,1,0,0,0,0,0,0,0,0,0,0,0,0,0,0,0,1,0,0,0,0,0,0,0,0,0,0,0,0,0,0,0,0,0,0,0,0,0,0,0,1,0,0,0,0,0,0,1,0,0,0,0,0,0,0,0,0,0,0

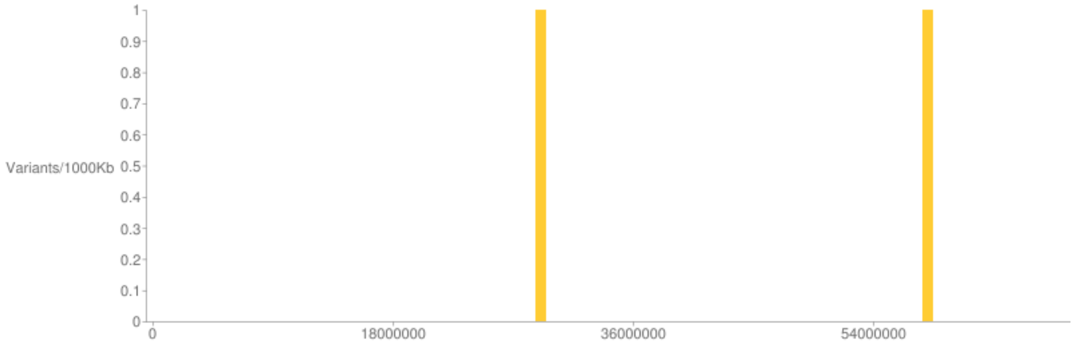

2, Position,0,1000000,2000000,3000000,4000000,5000000,6000000,7000000,8000000,9000000,10000000,11000000,12000000,130000
2,Count,0,0,0,0,0,0,0,0,0,0,0,0,0,0,0,0,0,0,0,0,0,0,0,0,0,0,0,0,0,1,0,0,0,0,0,0,0,0,0,0,0,0,0,0,0,0,0,0,0,0,0,0,0,0,0,0,0,0,1,0,0,0,0,0

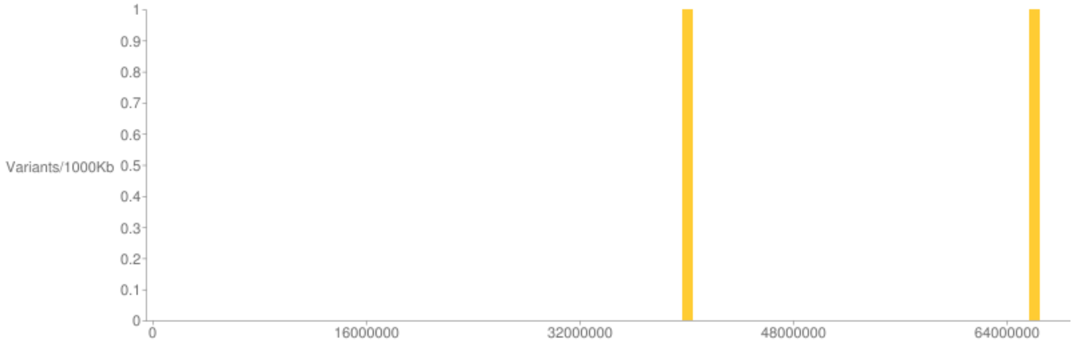

3, Position,0,1000000,2000000,3000000,4000000,5000000,6000000,7000000,8000000,9000000,10000000,11000000,12000000,130000
3,Count,0,0,0,0,0,0,0,0,0,0,0,0,0,0,0,0,0,0,0,0,0,0,0,0,0,0,0,0,0,0,0,0,0,0,0,0,0,0,0,0,1,0,0,0,0,0,0,0,0,0,0,0,0,0,0,0,0,0,0,0,0,0,0,0

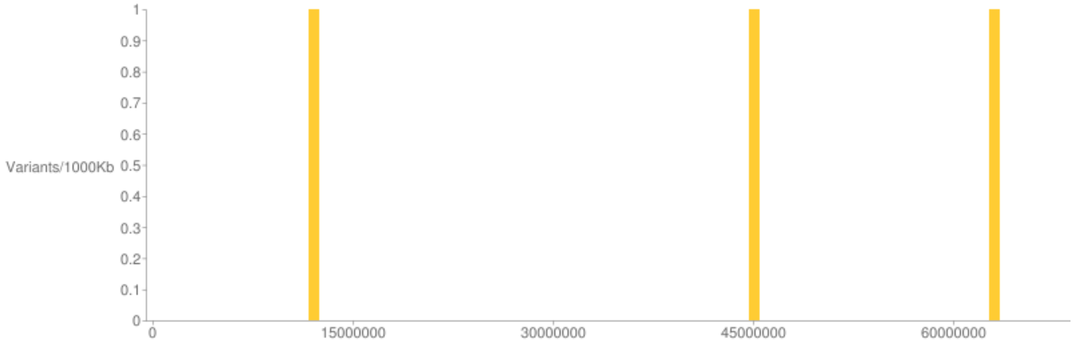

4, Position,0,1000000,2000000,3000000,4000000,5000000,6000000,7000000,8000000,9000000,10000000,11000000,12000000,130000
4,Count,0,0,0,0,0,0,0,0,0,0,0,0,1,0,0,0,0,0,0,0,0,0,0,0,0,0,0,0,0,0,0,0,0,0,0,0,0,0,0,0,0,0,0,0,0,1,0,0,0,0,0,0,0,0,0,0,0,0,0,0,0,0,0,1

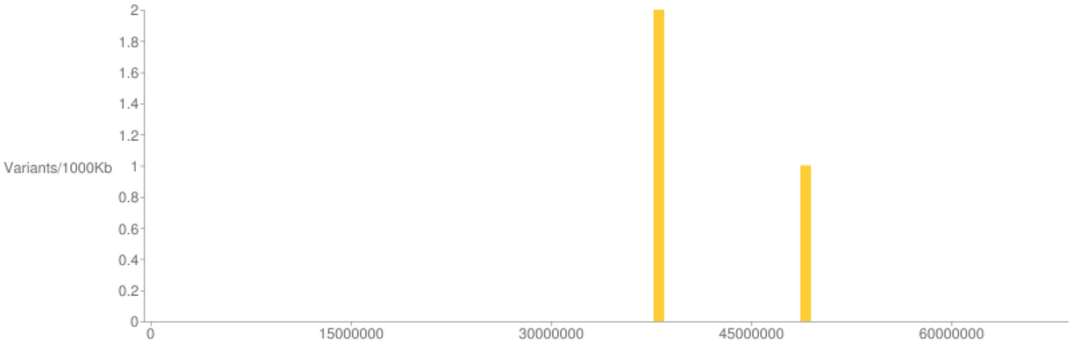

5, Position,0,1000000,2000000,3000000,4000000,5000000,6000000,7000000,8000000,9000000,10000000,11000000,12000000,130000
5,Count,0,0,0,0,0,0,0,0,0,0,0,0,0,0,0,0,0,0,0,0,0,0,0,0,0,0,0,0,0,0,0,0,0,0,0,0,0,0,2,0,0,0,0,0,0,0,0,0,0,1,0,0,0,0,0,0,0,0,0,0,0,0,0,0

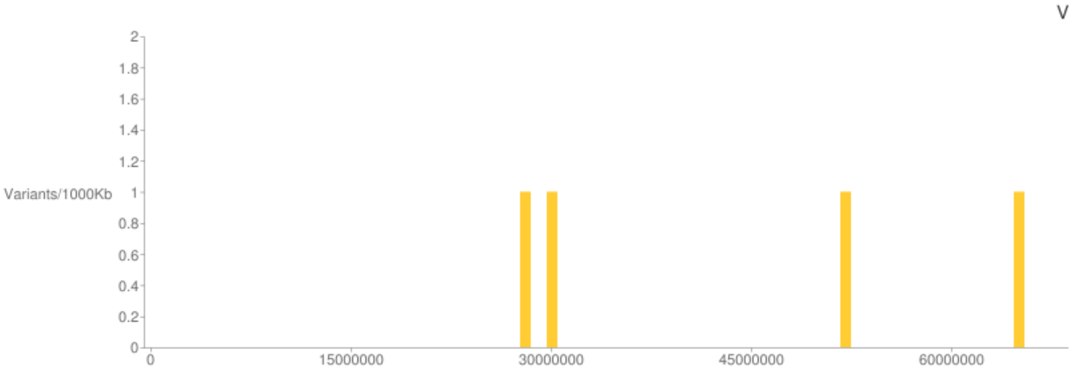

6, Position,0,1000000,2000000,3000000,4000000,5000000,6000000,7000000,8000000,9000000,10000000,11000000,12000000,130000
6,Count,0,0,0,0,0,0,0,0,0,0,0,0,0,0,0,0,0,0,0,0,0,0,0,0,0,0,0,0,1,0,1,0,0,0,0,0,0,0,0,0,0,0,0,0,0,0,0,0,0,0,0,0,1,0,0,0,0,0,0,0,0,0,0,0

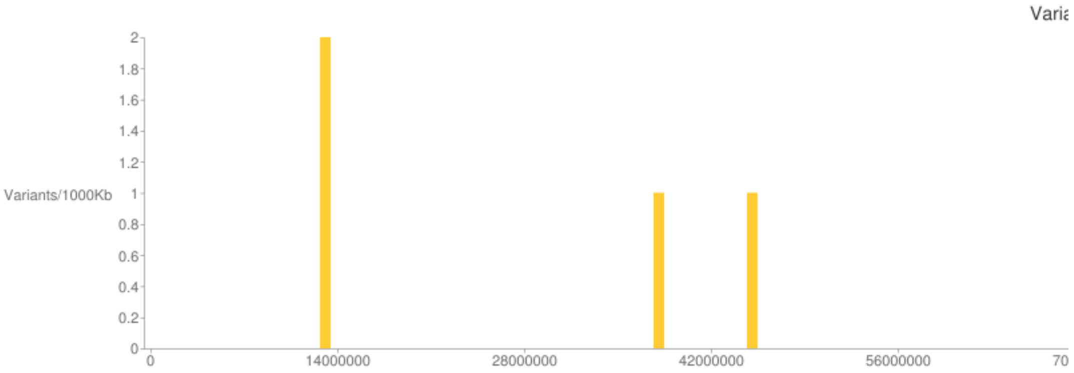

7, Position,0,1000000,2000000,3000000,4000000,5000000,6000000,7000000,8000000,9000000,10000000,11000000,12000000,130000
7,Count,0,0,0,0,0,0,0,0,0,0,0,0,0,2,0,0,0,0,0,0,0,0,0,0,0,0,0,0,0,0,0,0,0,0,0,0,0,0,1,0,0,0,0,0,0,1,0,0,0,0,0,0,0,0,0,0,0,0,0,0,0,0,0,0

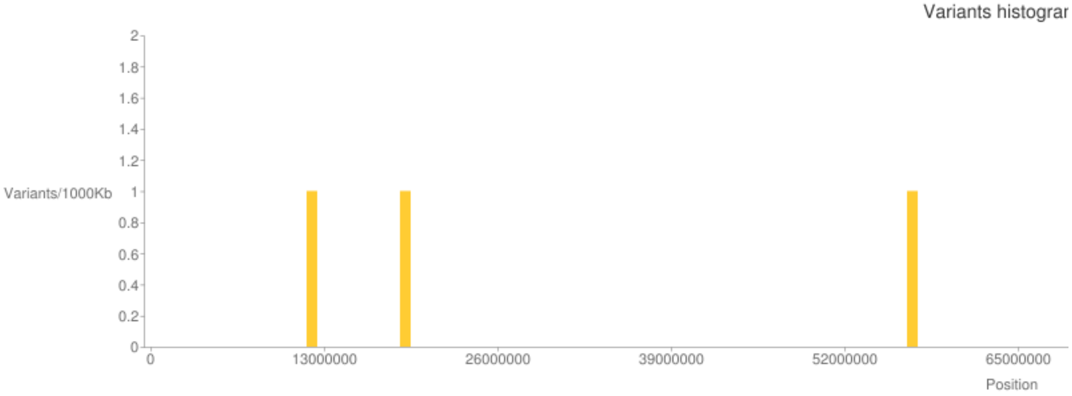

8, Position,0,1000000,2000000,3000000,4000000,5000000,6000000,7000000,8000000,9000000,10000000,11000000,12000000,130000
8,Count,0,0,0,0,0,0,0,0,0,0,0,0,1,0,0,0,0,0,0,1,0,0,0,0,0,0,0,0,0,0,0,0,0,0,0,0,0,0,0,0,0,0,0,0,0,0,0,0,0,0,0,0,0,0,0,0,0,1,0,0,0,0,0,0

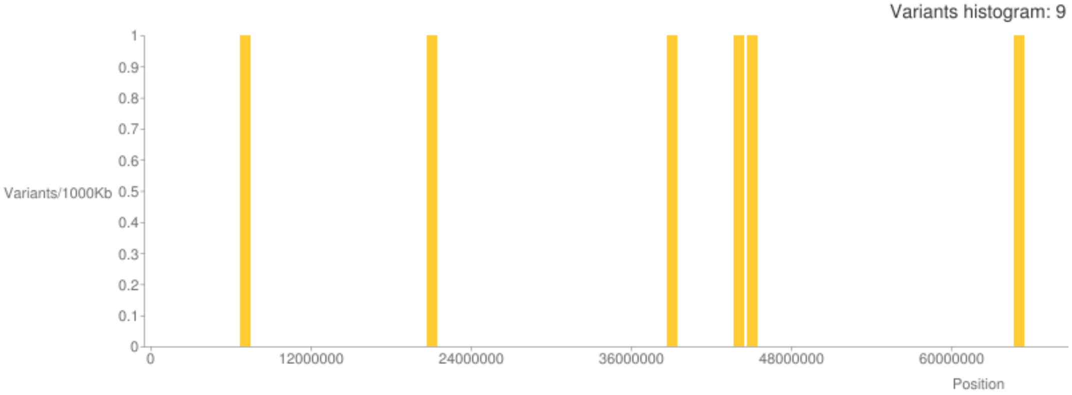

9, Position,0,1000000,2000000,3000000,4000000,5000000,6000000,7000000,8000000,9000000,10000000,11000000,12000000,130000
9,Count,0,0,0,0,0,0,0,1,0,0,0,0,0,0,0,0,0,0,0,0,0,1,0,0,0,0,0,0,0,0,0,0,0,0,0,0,0,0,0,1,0,0,0,0,1,1,0,0,0,0,0,0,0,0,0,0,0,0,0,0,0,0,0,0

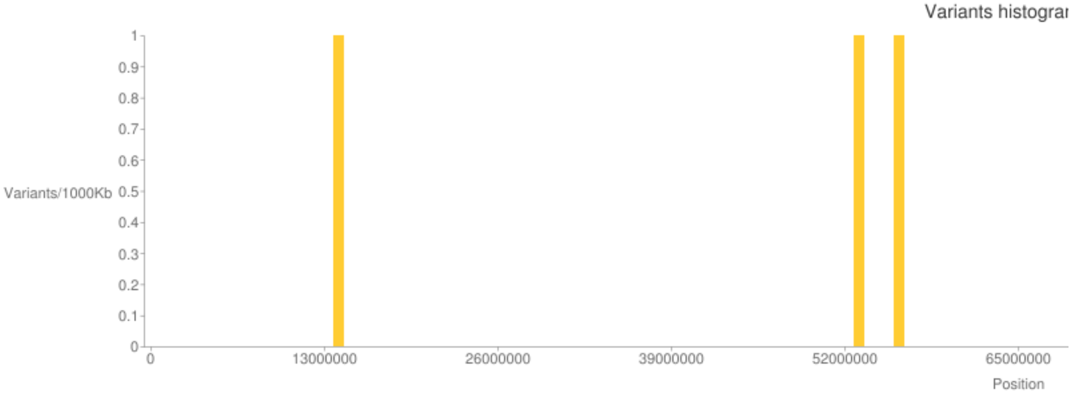

10, Position,0,1000000,2000000,3000000,4000000,5000000,6000000,7000000,8000000,9000000,10000000,11000000,12000000,13000
10,Count,0,0,0,0,0,0,0,0,0,0,0,0,0,0,1,0,0,0,0,0,0,0,0,0,0,0,0,0,0,0,0,0,0,0,0,0,0,0,0,0,0,0,0,0,0,0,0,0,0,0,0,0,0,1,0,0,1,0,0,0,0,0,0,

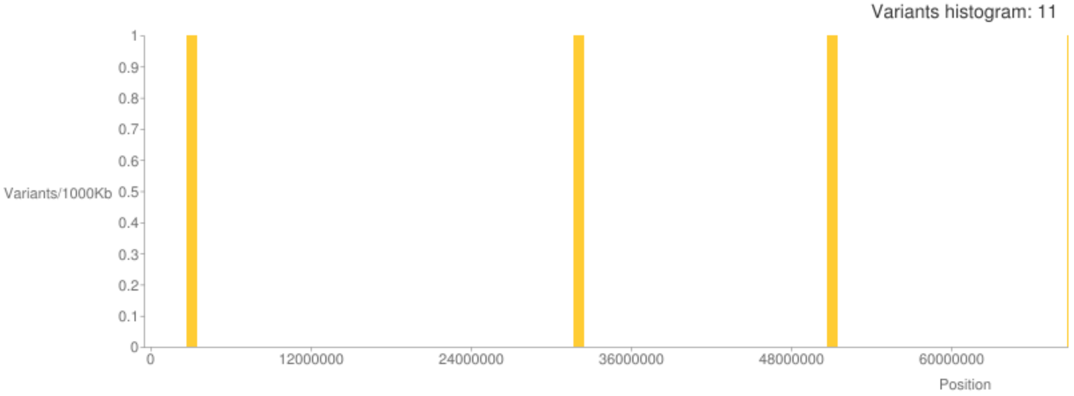

11, Position,0,1000000,2000000,3000000,4000000,5000000,6000000,7000000,8000000,9000000,10000000,11000000,12000000,13000
11,Count,0,0,0,1,0,0,0,0,0,0,0,0,0,0,0,0,0,0,0,0,0,0,0,0,0,0,0,0,0,0,0,0,1,0,0,0,0,0,0,0,0,0,0,0,0,0,0,0,0,0,0,0,1,0,0,0,0,0,0,0,0,0,0,

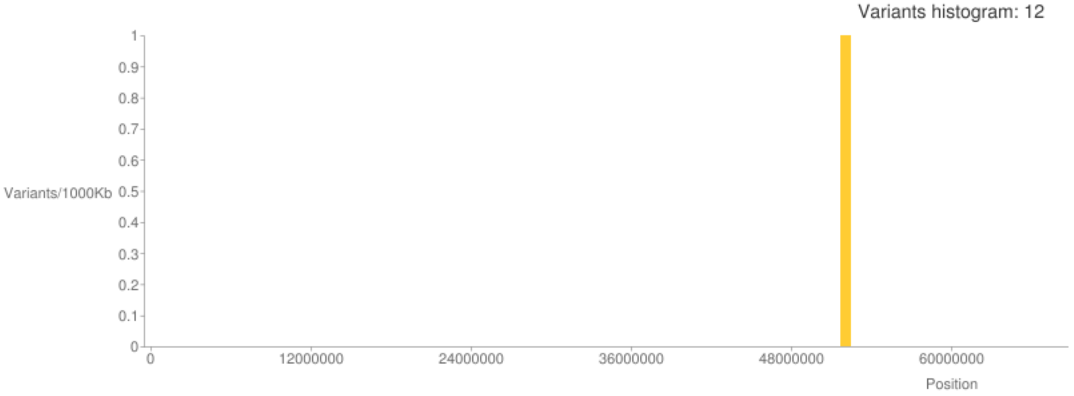

12, Position,0,1000000,2000000,3000000,4000000,5000000,6000000,7000000,8000000,9000000,10000000,11000000,12000000,13000
12,Count,0,0,0,0,0,0,0,0,0,0,0,0,0,0,0,0,0,0,0,0,0,0,0,0,0,0,0,0,0,0,0,0,0,0,0,0,0,0,0,0,0,0,0,0,0,0,0,0,0,0,0,0,1,0,0,0,0,0,0,0,0,0,0,

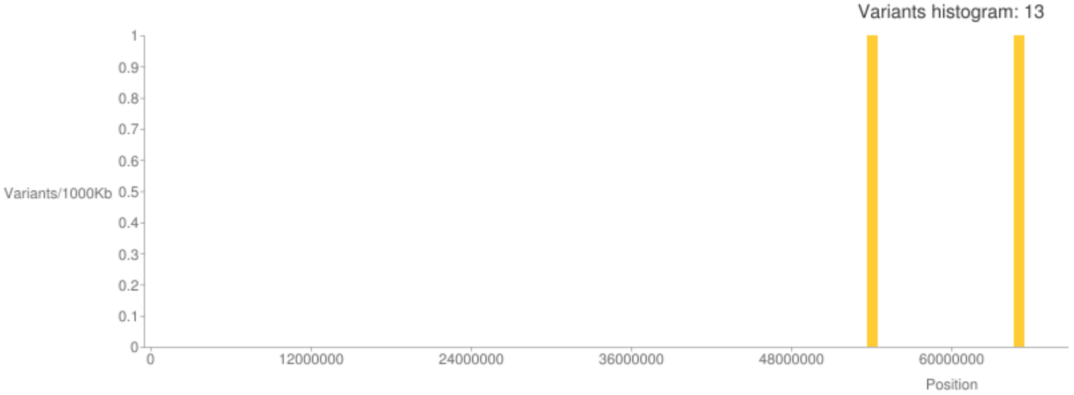

13, Position,0,1000000,2000000,3000000,4000000,5000000,6000000,7000000,8000000,9000000,10000000,11000000,12000000,13000
13,Count,0,0,0,0,0,0,0,0,0,0,0,0,0,0,0,0,0,0,0,0,0,0,0,0,0,0,0,0,0,0,0,0,0,0,0,0,0,0,0,0,0,0,0,0,0,0,0,0,0,0,0,0,0,0,1,0,0,0,0,0,0,0,0,

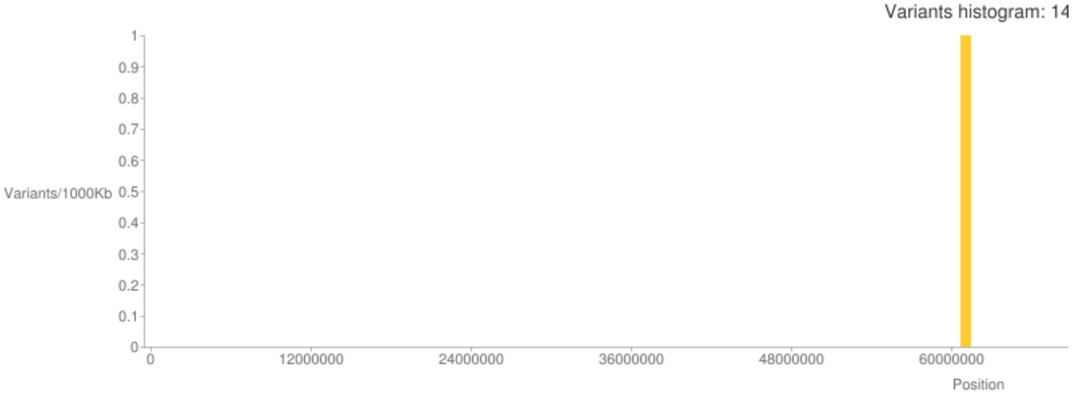

14, Position,0,1000000,2000000,3000000,4000000,5000000,6000000,7000000,8000000,9000000,10000000,11000000,12000000,13000
14,Count,0,0,0,0,0,0,0,0,0,0,0,0,0,0,0,0,0,0,0,0,0,0,0,0,0,0,0,0,0,0,0,0,0,0,0,0,0,0,0,0,0,0,0,0,0,0,0,0,0,0,0,0,0,0,0,0,0,0,0,0,0,1,0,

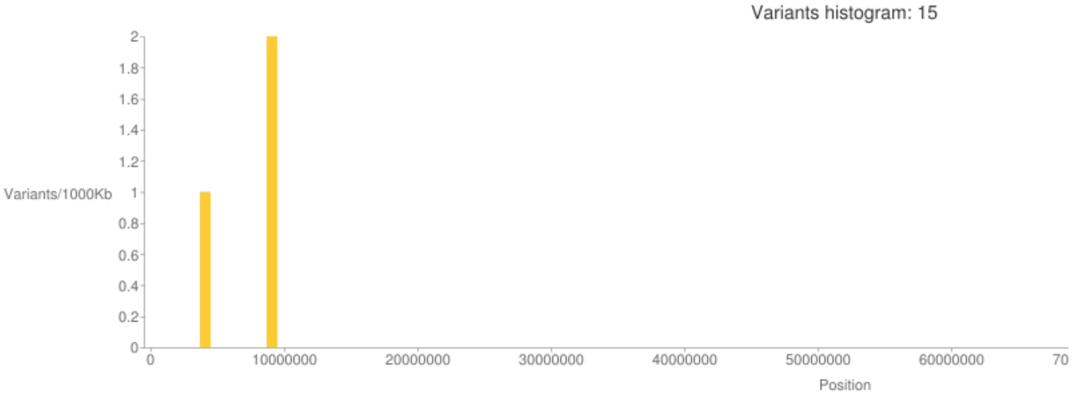

15, Position,0,1000000,2000000,3000000,4000000,5000000,6000000,7000000,8000000,9000000,10000000,11000000,12000000,13000
15,Count,0,0,0,0,1,0,0,0,0,2,0,0,0,0,0,0,0,0,0,0,0,0,0,0,0,0,0,0,0,0,0,0,0,0,0,0,0,0,0,0,0,0,0,0,0,0,0,0,0,0,0,0,0,0,0,0,0,0,0,0,0,0,0,

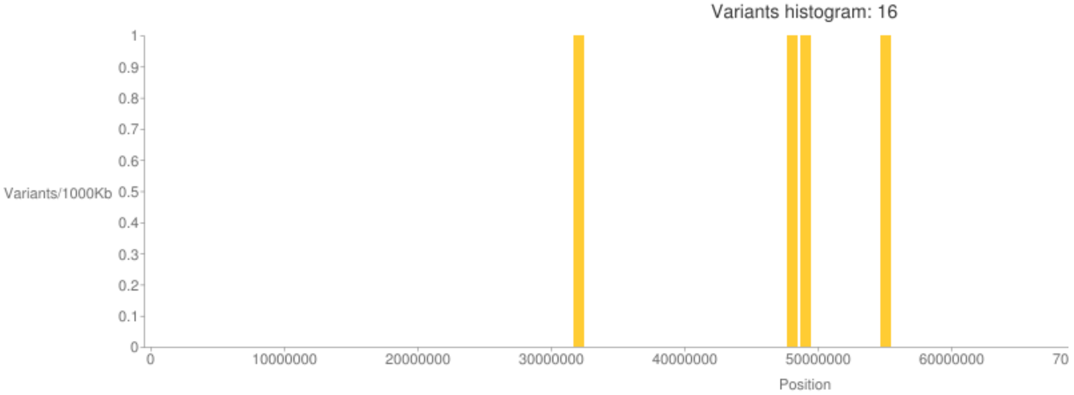

16, Position,0,1000000,2000000,3000000,4000000,5000000,6000000,7000000,8000000,9000000,10000000,11000000,12000000,13000
16,Count,0,0,0,0,0,0,0,0,0,0,0,0,0,0,0,0,0,0,0,0,0,0,0,0,0,0,0,0,0,0,0,0,1,0,0,0,0,0,0,0,0,0,0,0,0,0,0,0,1,1,0,0,0,0,0,1,0,0,0,0,0,0,0,

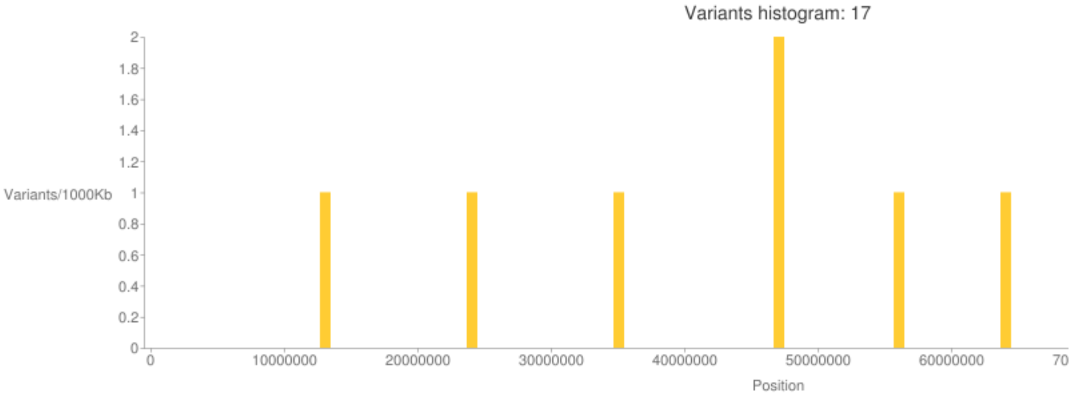

17, Position,0,1000000,2000000,3000000,4000000,5000000,6000000,7000000,8000000,9000000,10000000,11000000,12000000,13000
17,Count,0,0,0,0,0,0,0,0,0,0,0,0,0,1,0,0,0,0,0,0,0,0,0,0,1,0,0,0,0,0,0,0,0,0,0,1,0,0,0,0,0,0,0,0,0,0,0,2,0,0,0,0,0,0,0,0,1,0,0,0,0,0,0,

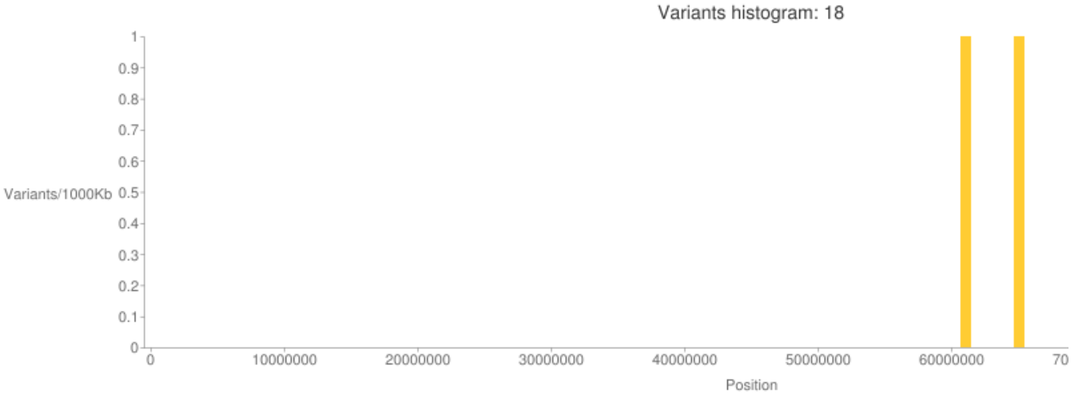

18, Position,0,1000000,2000000,3000000,4000000,5000000,6000000,7000000,8000000,9000000,10000000,11000000,12000000,13000
18,Count,0,0,0,0,0,0,0,0,0,0,0,0,0,0,0,0,0,0,0,0,0,0,0,0,0,0,0,0,0,0,0,0,0,0,0,0,0,0,0,0,0,0,0,0,0,0,0,0,0,0,0,0,0,0,0,0,0,0,0,0,0,1,0,

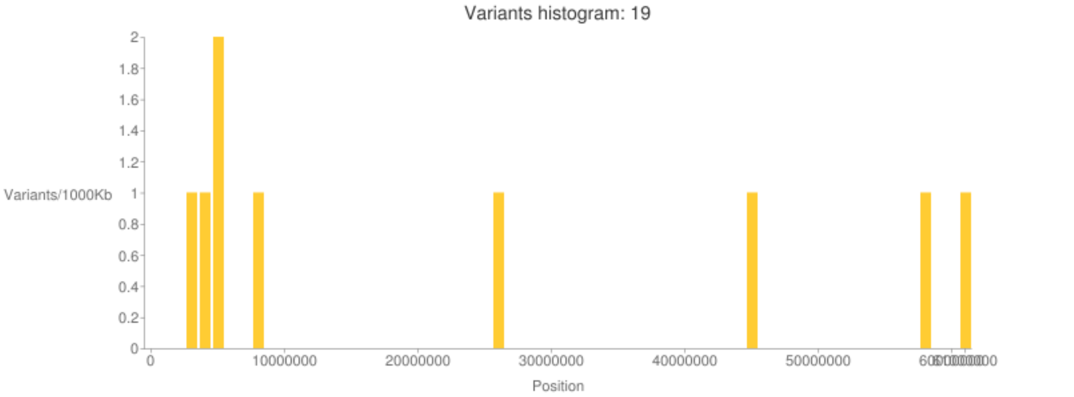

19, Position,0,1000000,2000000,3000000,4000000,5000000,6000000,7000000,8000000,9000000,10000000,11000000,12000000,13000
19,Count,0,0,0,1,1,2,0,0,1,0,0,0,0,0,0,0,0,0,0,0,0,0,0,0,0,0,1,0,0,0,0,0,0,0,0,0,0,0,0,0,0,0,0,0,0,0,1,0,0,0,0,0,0,0,0,0,0,0,0,1,0,0,1

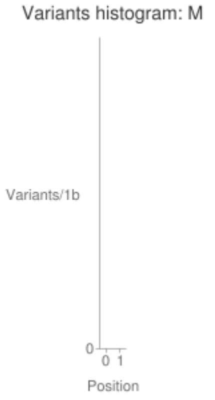

M, Position,0,1
M,Count,0,0

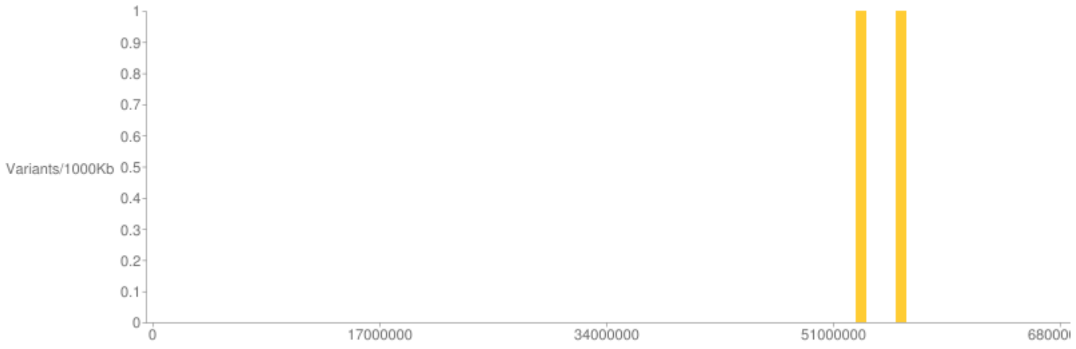

X, Position,0,1000000,2000000,3000000,4000000,5000000,6000000,7000000,8000000,9000000,10000000,11000000,12000000,130000
X,Count,0,0,0,0,0,0,0,0,0,0,0,0,0,0,0,0,0,0,0,0,0,0,0,0,0,0,0,0,0,0,0,0,0,0,0,0,0,0,0,0,0,0,0,0,0,0,0,0,0,0,0,0,0,1,0,0,1,0,0,0,0,0,0,0

Details by gene

Here you can find a tab-separated table.

SnpEff: Variant analysis

Contents

Summary
Variant rate by chromosome
Variants by type
Number of variants by impact
Number of variants by functional class
Number of variants by effect
Quality histogram
InDel length histogram
Base variant table
Transition vs transversions (ts/tv)
Allele frequency
Allele Count
Codon change table
Amino acid change table
Chromosome variants plots
Details by gene

Summary

**Genome**	GRCm38.75
**Date**	2018-06-04 16:52
**SnpEff version**	SnpEff 4.3t (build 2017-11-24 10:18), by Pablo Cingolani
**Command line arguments**	SnpEff -stats ../output/SnpEff/RP-mSCC1-P30-_SNP_snpEff_summary.html -no-intergenic -no-intron GRCm38.75 ../output/Variants_Filtered/RP-mSCC1-P30-_SNP_PASS.vcf
**Warnings**	87
**Errors**	0
**Number of lines (input file)**	3,403
**Number of variants (before filter)**	3,404
**Number of not variants (i.e. reference equals alternative)**	0
**Number of variants processed (i.e. after filter and non-variants)**	3,404
**Number of known variants (i.e. non-empty ID)**	105 (3.085%)
**Number of multi-allelic VCF entries (i.e. more than two alleles)**	1
**Number of effects**	4,610
**Genome total length**	4,343,312,699
**Genome effective length**	2,633,776,672
**Variant rate**	1 variant every 773,729 bases

Variants rate details

Chromosome	Length	Variants	Variants rate
1	195,471,971	353	553,744
2	182,113,224	294	619,432
3	160,039,680	156	1,025,895
4	156,508,116	146	1,071,973
5	151,834,684	189	803,358
6	149,736,546	255	587,202
7	145,441,459	238	611,098
8	129,401,213	194	667,016
9	124,595,110	189	659,233
10	130,694,993	176	742,585
11	122,082,543	285	428,359
12	120,129,022	96	1,251,343
13	120,421,639	82	1,468,556
14	124,902,244	128	975,798
15	104,043,685	108	963,367
16	98,207,768	63	1,558,853
17	94,987,271	110	863,520
18	90,702,639	74	1,225,711
19	61,431,566	107	574,126
X	171,031,299	161	1,062,306
**Total**	**2,633,776,672**	**3,404**	**773,729**

Number variants by type

Type	Total
**SNP**	3,404
**MNP**	0
**Total**	**3,404**

Type	Total
INS	0
DEL	0
MIXED	0
INV	0
DUP	0
BND	0
INTERVAL	0
Total	3,404

Number of effects by impact

Type (alphabetical order)	Count	Percent
HIGH	176	3.818%
LOW	767	16.638%
MODERATE	1,727	37.462%
MODIFIER	1,940	42.082%

Number of effects by functional class

Type (alphabetical order)	Count	Percent
MISSENSE	1,730	69.871%
NONSENSE	137	5.533%
SILENT	609	24.596%

Missense / Silent ratio: 2.8407

Number of effects by type and region

Type

Type (alphabetical order)	Count	Percent
3_prime_UTR_variant	62	1.273%
5_prime_UTR_premature_start_codon_gain_variant	11	0.226%
5_prime_UTR_variant	46	0.945%
downstream_gene_variant	437	8.973%
intergenic_region	44	0.903%
intragenic_variant	77	1.581%
intron_variant	1,042	21.396%
missense_variant	1,727	35.462%
non_coding_transcript_exon_variant	82	1.684%
splice_acceptor_variant	20	0.411%
splice_donor_variant	16	0.329%
splice_region_variant	226	4.641%
start_lost	2	0.041%
stop_gained	137	2.813%
stop_lost	1	0.021%
stop_retained_variant	1	0.021%
synonymous_variant	608	12.485%
upstream_gene_variant	331	6.797%

Region

Type (alphabetical order)	Count	Percent
DOWNSTREAM	437	9.479%
EXON	2,542	55.141%
INTERGENIC	44	0.954%
INTRON	862	18.698%
SPLICE_SITE_ACCEPTOR	20	0.434%
SPLICE_SITE_DONOR	16	0.347%
SPLICE_SITE_REGION	162	3.514%
TRANSCRIPT	77	1.67%
UPSTREAM	331	7.18%
UTR_3_PRIME	62	1.345%
UTR_5_PRIME	57	1.236%

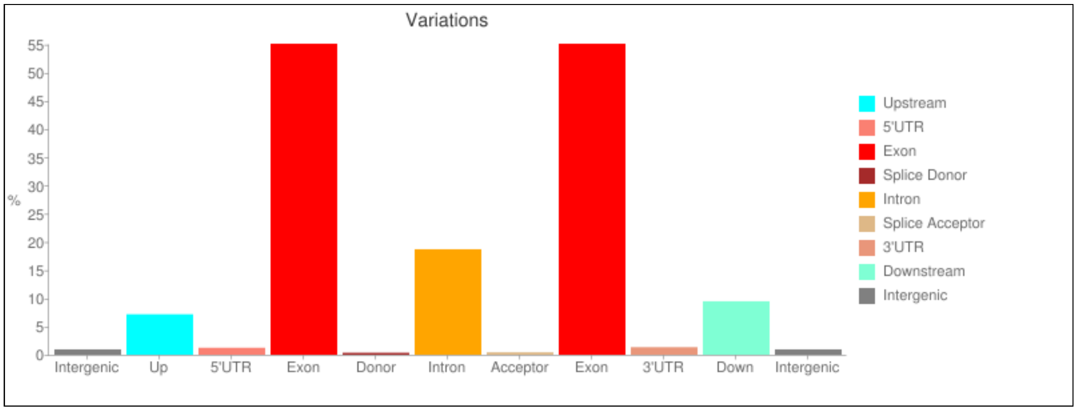

Quality:

Min	104
Max	120,408
Mean	807.636

**Median**	541
**Standard deviation**	2,343.043
**Values**	104,106,111,115,116,119,120,121,122,123,124,126,127,129,130,131,137,138,139,140,141,142,143,144,145,146,147,149,150,151,152,153,154,156,157
**Count**	1,1,1,1,1,1,1,1,1,1,2,3,6,1,3,3,1,1,1,1,4,2,1,3,2,1,1,1,3,1,3,2,3,1,1,2,1,2,1,3,1,2,3,3,1,3,2,1,3,2,3,1,2,2,1,4,2,1,3,4,4,4,2,2,3,1,3,2,5,5,1,2,1,2,3,2,1,2,2,5,4,

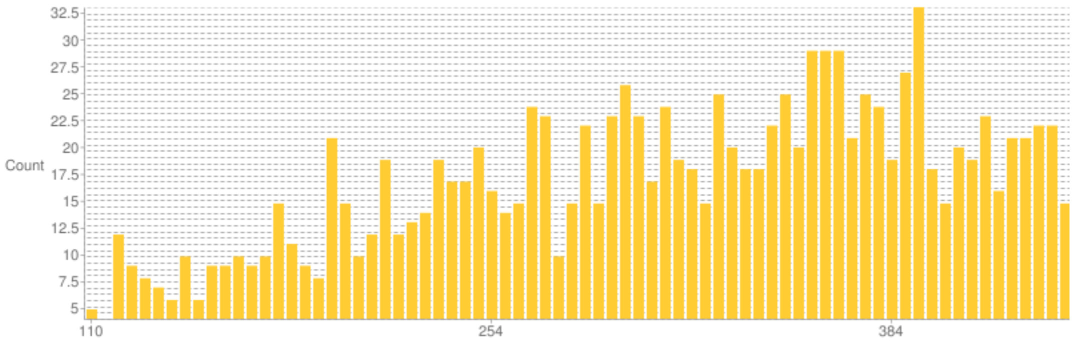

Insertions and deletions length:

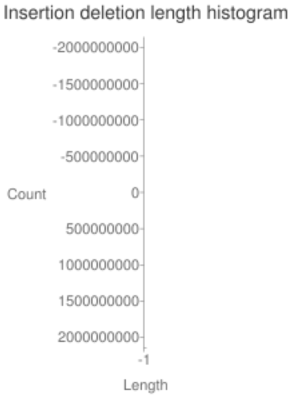

Base changes (SNPs)

	A	C	G	T
**A**	0	14	41	17
**C**	975	0	202	430
**G**	410	179	0	1,047
**T**	23	54	12	0

Ts/Tv (transitions / transversions)

Note: Only SNPs are used for this statistic.
Note: This Ts/Tv ratio is a 'raw' ratio (ratio of observed events).

**Transitions**	1,015
**Transversions**	2,567
**Ts/Tv ratio**	0.3954

All variants:

```
Sample ,RS-03044064,Total
Transitions ,1015,1015
Transversions ,2567,2567
Ts/Tv ,0.395,0.395
```

Only known variants (i.e. the ones having a non-empty ID field):

```
Sample ,RS-03044064,Total
Transitions ,125,125
Transversions ,50,50
Ts/Tv ,2.500,2.500
```

Allele frequency

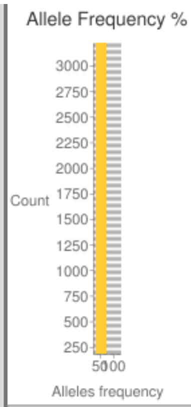

**Min**	50
**Max**	100
**Mean**	52.659
**Median**	50
**Standard deviation**	11.222
**Values**	50,100
**Count**	3222,181

Allele Count

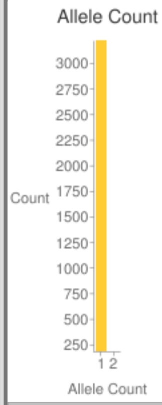

**Min**	1
**Max**	2
**Mean**	1.053
**Median**	1
**Standard deviation**	0.224
**Values**	1,2
**Count**	3222,181

Hom/Het per sample

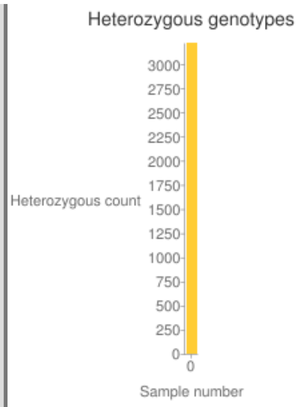

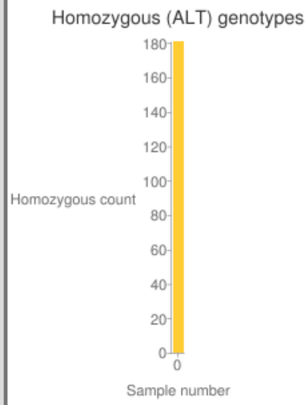

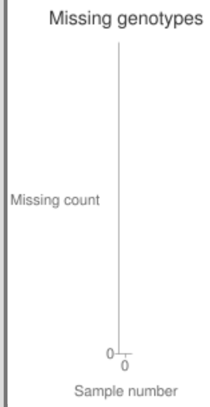


```
Sample_names , RS-03044064
Reference , 0
Het , 3222
Hom , 181
Missing , 0
```

Codon changes

How to read this table:
- Rows are reference codons and columns are changed codons. E.g. Row 'AAA' column 'TAA' indicates how many 'AAA' codons have been replaced by 'TAA' codons.
- Red background colors indicate that more changes happened (heat-map).
- Diagonals are indicated using grey background color
- WARNING: This table may include different translation codon tables (e.g. mamalian DNA and mitochondrial DNA).

	AAA	AAC	AAG	AAT	ACA	ACC	ACG	ACT	AGA	AGC	AGG	AGT	ATA	ATC	ATG	ATT	CAA	CAC	CAG	CAT	CCA	CCC	CCG
AAA			1						1														
AAC	17		2	8		1								1									
AAG	17	3		45			1				1				1				2				
AAT	1											1											
ACA	13						1		5				7										
ACC		11			26		1	6		3				3									
ACG			6		9	1		11			2				13								
ACT				6		1						2				3							
AGA	2										2		9										
AGC		5				1			18		3	6		17									
AGG			7				2		8			10			19								
AGT				3				2	1	2						16							

	AAA	AAC	AAG	AAT	ACA	ACC	ACG	ACT	AGA	AGC	AGG	AGT	ATA	ATC	ATG	ATT	CAA	CAC	CAG	CAT	CCA	CCC	CCG
ATA	1														1	1							
ATC													10			13							
ATG			1				1						16	2		31							
ATT								2						1									
CAA	4																				2		
CAC		7															10		13	5			
CAG			26														14	5		40			
CAT				12														1					
CCA					15												13					1	
CCC						9												19			20		4
CCG							5												9		7	1	
CCT								14												15	1	1	
CGA									5								3						
CGC										22								12				1	
CGG											13								7				13
CGT												8								5			
CTA													4										
CTC														8								1	
CTG															29				1				3
CTT																10				1			
GAA	10																4						
GAC		8																7					
GAG			22																9				
GAT				17																3			
GCA					2																3		
GCC						3																2	
GCG							2																1
GCT								8															
GGA									17														
GGC										17													
GGG											9												
GGT												4											
GTA													2										
GTC														2									
GTG															11								
GTT																4							
TAA	1																						
TAC		1																					
TAG																							
TAT																							
TCA					1																		
TCC						1																	
TCG																							
TCT																							
TGA																							
TGC																							
TGG																							
TGT																							
TTA																							
TTC																							
TTG																							
TTT															1								

Amino acid changes

How to read this table:
- Rows are reference amino acids and columns are changed amino acids. E.g. Row 'A' column 'E' indicates how many 'A' amino acids have been replaced by 'E' amino acids.
- Red background colors indicate that more changes happened (heat-map).
- Diagonals are indicated using grey background color
- WARNING: This table may include different translation codon tables (e.g. mamalian DNA and mitochondrial DNA).

	*	A	C	D	E	F	G	H	I	K	L	M	N	P	Q	R	S	T	V	W	Y
*	1									1											
A		69		29	14		5							8			53	15	16		
C	12		4			26										1	6			1	5
D		1		6	17		1	10					25								38
E	60			26	21		1			32					13						
F						6			1		20						2				

	*	A	C	D	E	F	G	H	I	K	L	M	N	P	Q	R	S	T	V	W	Y
G	23	16	53	7	15		42									51	21		73	30	
H				1				6					19	1	23	1					5
I						1			25	1		1						2	3		
K					1					18		1	48		2	2		1			
L						24		1	22		100	29		4	1				9		
M									49	1								1			
N				1					1	20			8				1	1			1
P		7						34			35			53	22	16	13	43			
Q	6				7			45		30				2	14						
R	2		19				5	17	9	9	73	19		14	10	59	40	2		7	
S	8	1	3			16			33		5		8	1		23	64	5		1	21
T		1							13	19		13	17			7	5	56			
V		2				22			8		59	11							51		
W	12		15								15						4				
Y	14		1			10							1								6

Variants by chromosome

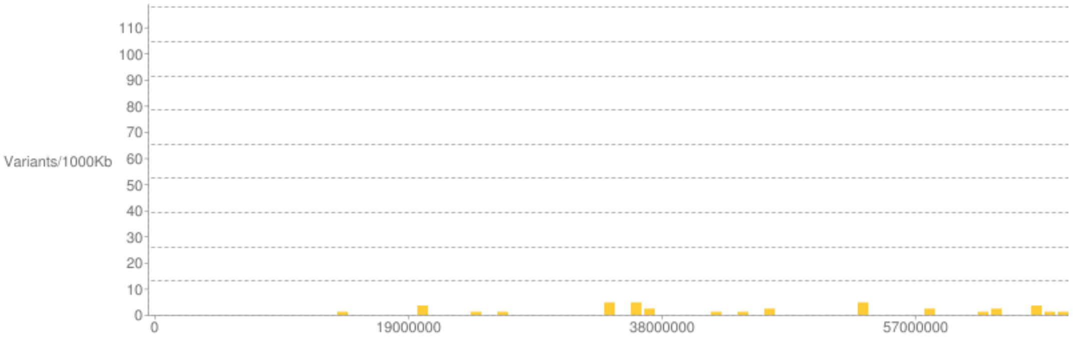

1, Position,0,1000000,2000000,3000000,4000000,5000000,6000000,7000000,8000000,9000000,10000000,11000000,12000000,130000
1,Count,0,0,0,0,1,0,1,0,0,1,0,1,1,0,2,0,0,0,0,0,4,0,0,0,2,1,2,0,1,0,1,1,0,1,5,0,5,3,0,1,1,0,2,1,2,0,3,0,0,0,0,0,1,5,1,1,0,0,3,1,1,0,2,3

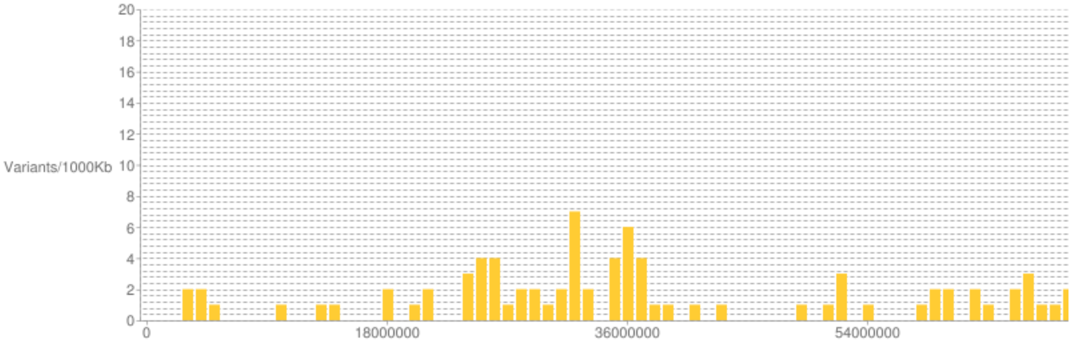

2, Position,0,1000000,2000000,3000000,4000000,5000000,6000000,7000000,8000000,9000000,10000000,11000000,12000000,130000
2,Count,0,0,0,2,2,1,0,0,0,0,1,0,0,1,1,0,0,0,2,0,1,2,0,0,3,4,4,1,2,2,1,2,7,2,0,4,6,4,1,1,0,1,0,1,0,0,0,0,0,0,1,0,1,3,0,1,0,0,0,1,2,2,0,2,1

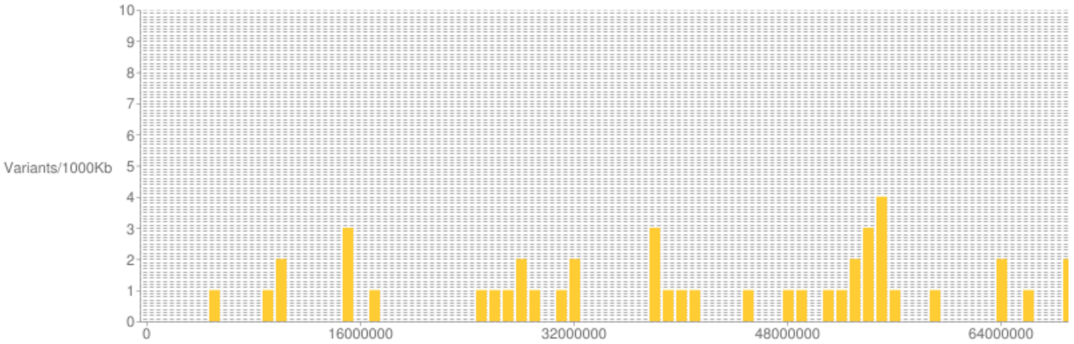

3, Position,0,1000000,2000000,3000000,4000000,5000000,6000000,7000000,8000000,9000000,10000000,11000000,12000000,130000
3,Count,0,0,0,0,0,1,0,0,0,1,2,0,0,0,0,3,0,1,0,0,0,0,0,0,0,1,1,1,2,1,0,1,2,0,0,0,0,0,3,1,1,1,0,0,0,1,0,0,1,1,0,1,1,2,3,4,1,0,0,1,0,0,0,0

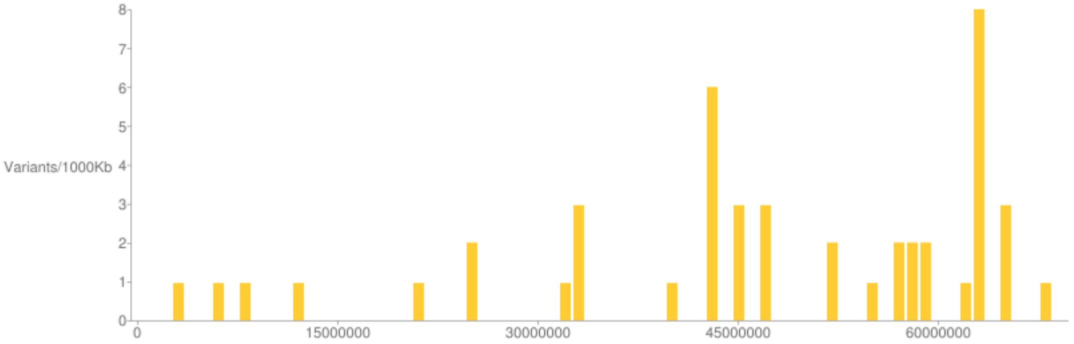

4, Position,0,1000000,2000000,3000000,4000000,5000000,6000000,7000000,8000000,9000000,10000000,11000000,12000000,130000
4,Count,0,0,0,1,0,0,1,0,1,0,0,0,1,0,0,0,0,0,0,0,1,0,0,0,2,0,0,0,0,0,0,1,3,0,0,0,0,0,0,1,0,0,6,0,3,0,3,0,0,0,0,2,0,0,1,0,2,2,2,0,0,1,8

V

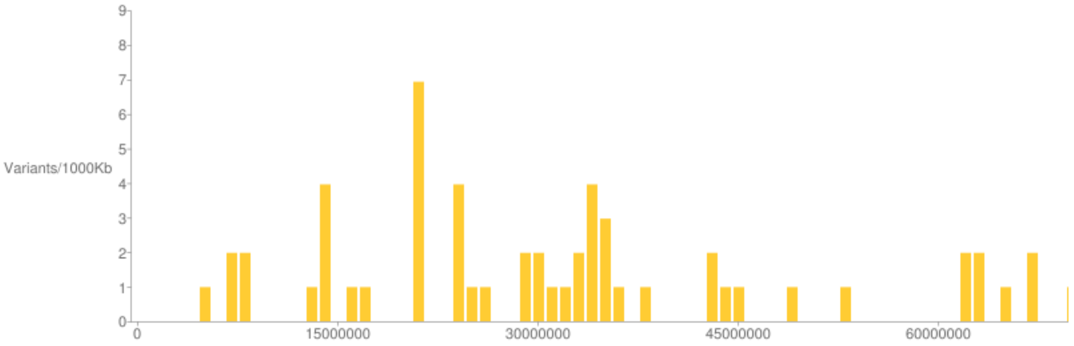

5, Position,0,1000000,2000000,3000000,4000000,5000000,6000000,7000000,8000000,9000000,10000000,11000000,12000000,130000
5,Count,0,0,0,0,0,1,0,2,2,0,0,0,0,1,4,0,1,1,0,0,0,7,0,0,4,1,1,0,0,2,2,1,1,2,4,3,1,0,1,0,0,0,0,2,1,1,0,0,0,1,0,0,0,1,0,0,0,0,0,0,0,0,2,2

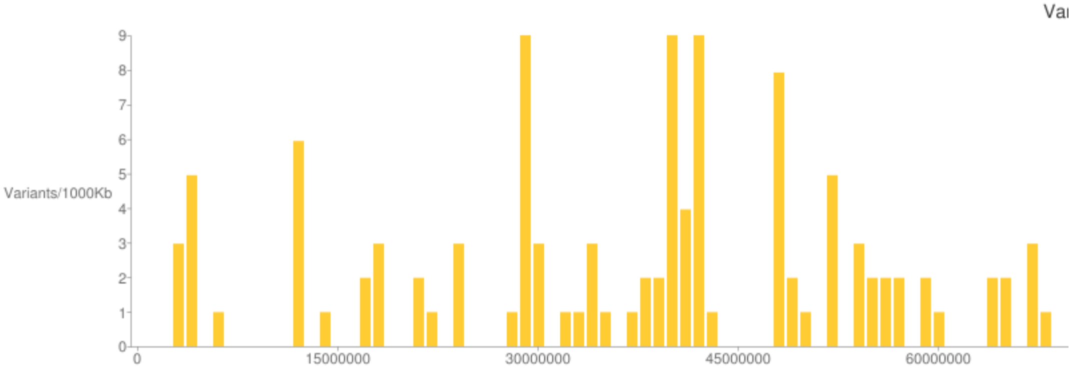

6, Position,0,1000000,2000000,3000000,4000000,5000000,6000000,7000000,8000000,9000000,10000000,11000000,12000000,130000
6,Count,0,0,0,3,5,0,1,0,0,0,0,0,6,0,1,0,0,2,3,0,0,2,1,0,3,0,0,0,1,9,3,0,1,1,3,1,0,1,2,2,9,4,9,1,0,0,0,0,8,2,1,0,5,0,3,2,2,2,0,2,1,0,0,0

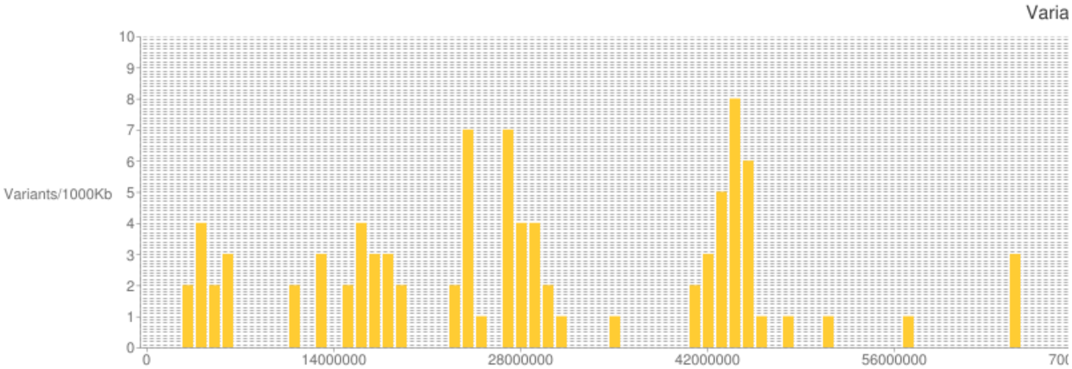

7, Position,0,1000000,2000000,3000000,4000000,5000000,6000000,7000000,8000000,9000000,10000000,11000000,12000000,130000
7,Count,0,0,0,2,4,2,3,0,0,0,0,2,0,3,0,2,4,3,3,2,0,0,0,2,7,1,0,7,4,4,2,1,0,0,0,1,0,0,0,0,0,2,3,5,8,6,1,0,1,0,0,1,0,0,0,0,0,1,0,0,0,0,0,0

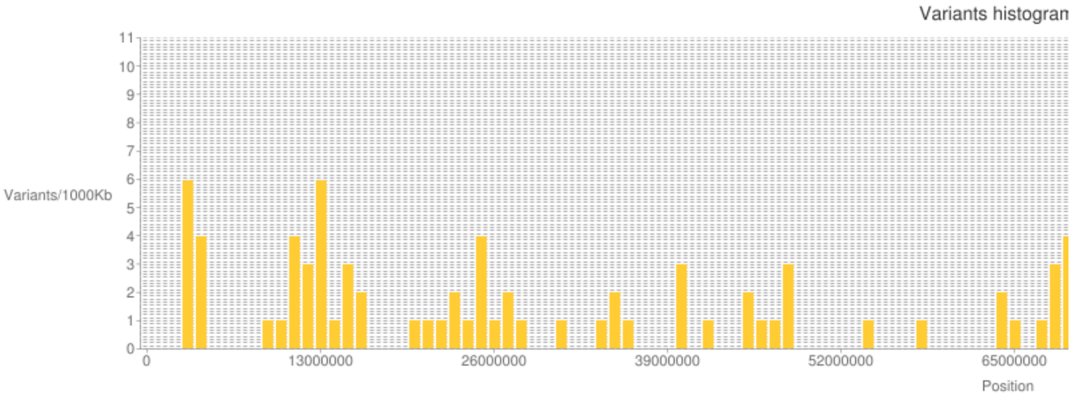

8, Position,0,1000000,2000000,3000000,4000000,5000000,6000000,7000000,8000000,9000000,10000000,11000000,12000000,130000
8,Count,0,0,0,6,4,0,0,0,0,1,1,4,3,6,1,3,2,0,0,0,1,1,1,2,1,4,1,2,1,0,0,1,0,0,1,2,1,0,0,0,3,0,1,0,0,2,1,1,3,0,0,0,0,0,1,0,0,0,1,0,0,0,0,0

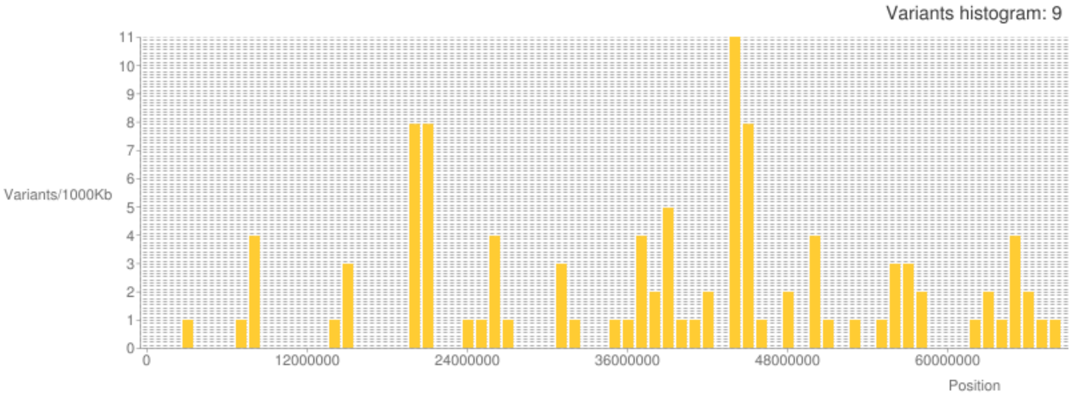

9, Position,0,1000000,2000000,3000000,4000000,5000000,6000000,7000000,8000000,9000000,10000000,11000000,12000000,130000
9,Count,0,0,0,1,0,0,0,1,4,0,0,0,0,0,1,3,0,0,0,0,8,8,0,0,1,1,4,1,0,0,0,3,1,0,0,1,1,4,2,5,1,1,2,0,11,8,1,0,2,0,4,1,0,1,0,1,3,3,2,0,0,0,1,

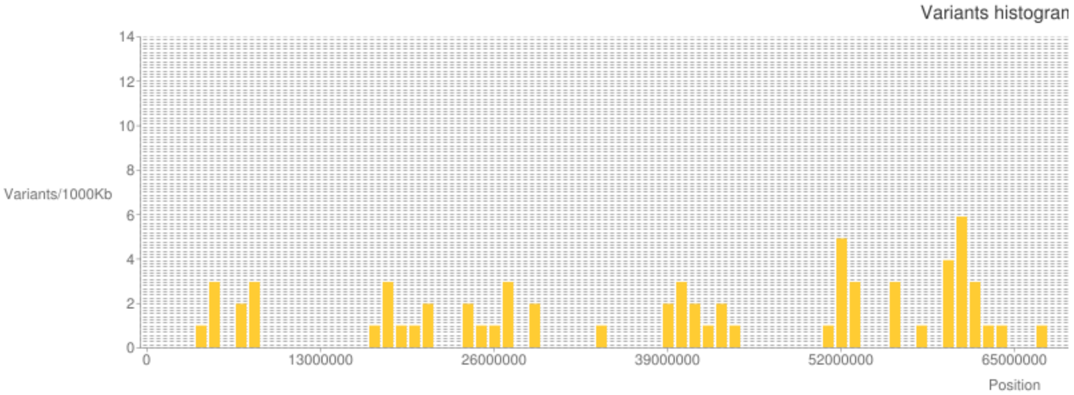

10, Position,0,1000000,2000000,3000000,4000000,5000000,6000000,7000000,8000000,9000000,10000000,11000000,12000000,13000
10,Count,0,0,0,0,1,3,0,2,3,0,0,0,0,0,0,0,1,3,1,1,2,0,0,2,1,1,3,0,2,0,0,0,0,1,0,0,0,0,2,3,2,1,2,1,0,0,0,0,0,0,1,5,3,0,0,3,0,1,0,4,6,3,

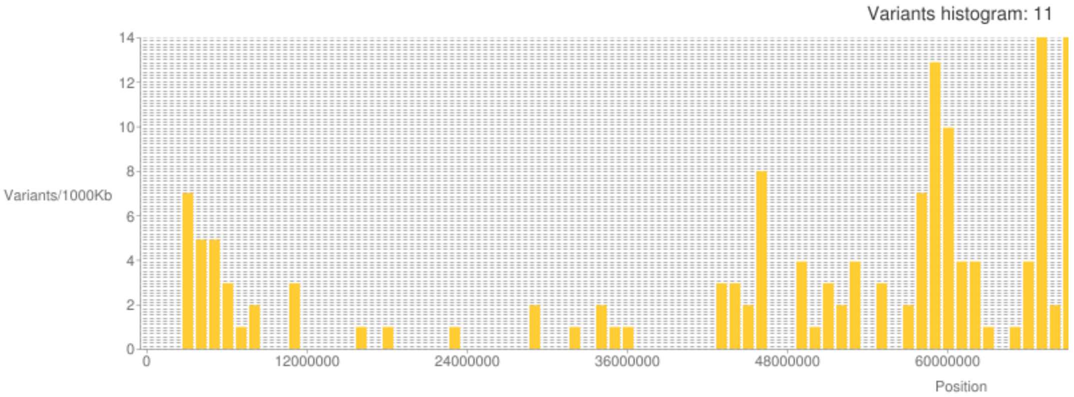

11, Position,0,1000000,2000000,3000000,4000000,5000000,6000000,7000000,8000000,9000000,10000000,11000000,12000000,13000
11,Count,0,0,0,7,5,5,3,1,2,0,0,3,0,0,0,0,1,0,1,0,0,0,0,1,0,0,0,0,0,2,0,0,1,0,2,1,1,0,0,0,0,0,0,3,3,2,8,0,0,4,1,3,2,4,0,3,0,2,7,13,10,4,

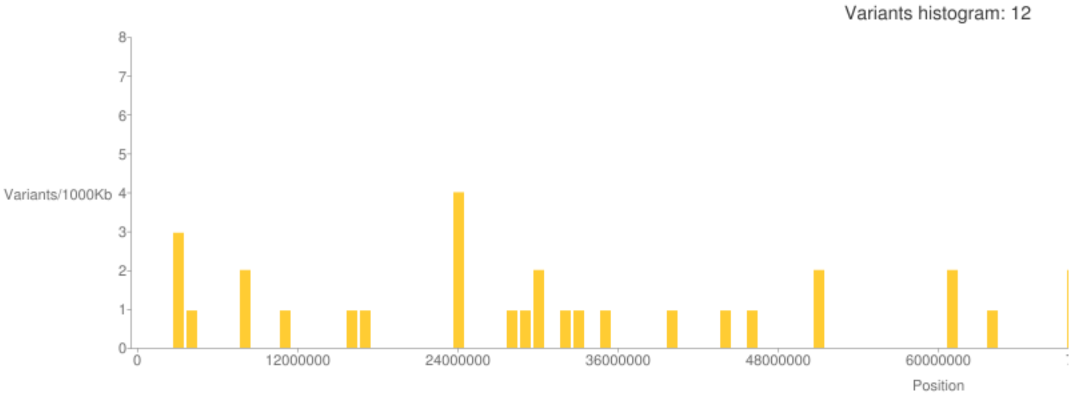

12, Position,0,1000000,2000000,3000000,4000000,5000000,6000000,7000000,8000000,9000000,10000000,11000000,12000000,13000
12,Count,0,0,0,3,1,0,0,0,2,0,0,1,0,0,0,0,1,1,0,0,0,0,0,0,4,0,0,0,1,1,2,0,1,1,0,1,0,0,0,0,1,0,0,0,1,0,1,0,0,0,0,2,0,0,0,0,0,0,0,0,0,2,0,

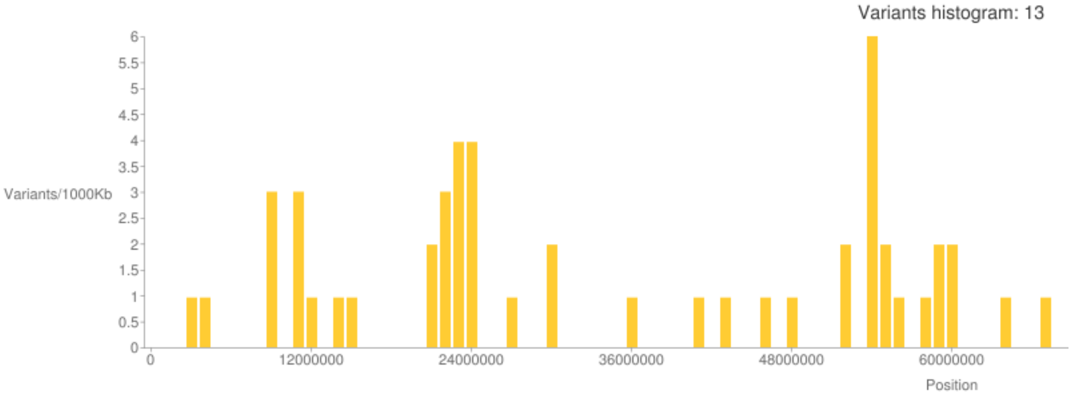

13, Position,0,1000000,2000000,3000000,4000000,5000000,6000000,7000000,8000000,9000000,10000000,11000000,12000000,13000
13,Count,0,0,0,1,1,0,0,0,0,3,0,3,1,0,1,1,0,0,0,0,0,2,3,4,4,0,0,1,0,0,2,0,0,0,0,0,1,0,0,0,0,1,0,1,0,0,1,0,1,0,0,0,2,0,6,2,1,0,1,2,2,0,0,

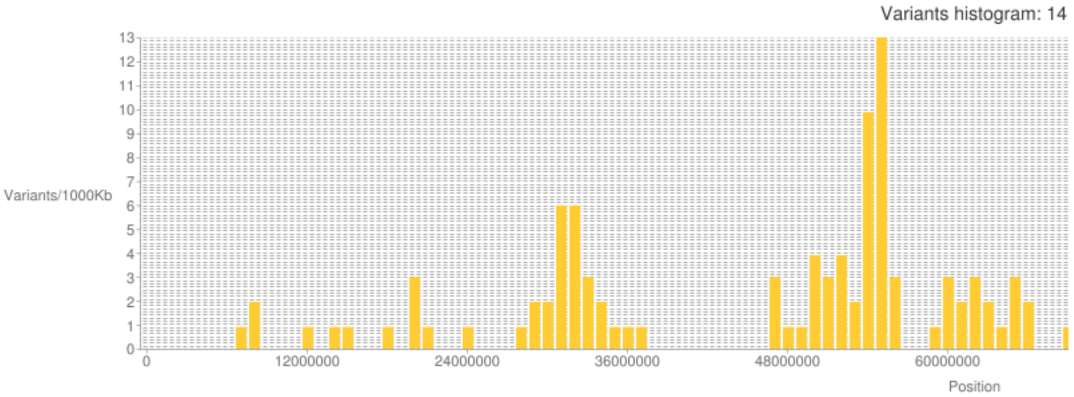

14, Position,0,1000000,2000000,3000000,4000000,5000000,6000000,7000000,8000000,9000000,10000000,11000000,12000000,13000
14,Count,0,0,0,0,0,0,0,1,2,0,0,0,1,0,1,1,0,0,1,0,3,1,0,0,1,0,0,0,1,2,2,6,6,3,2,1,1,1,0,0,0,0,0,0,0,0,3,1,1,4,3,4,2,10,13,3,0,0,1,3,2,

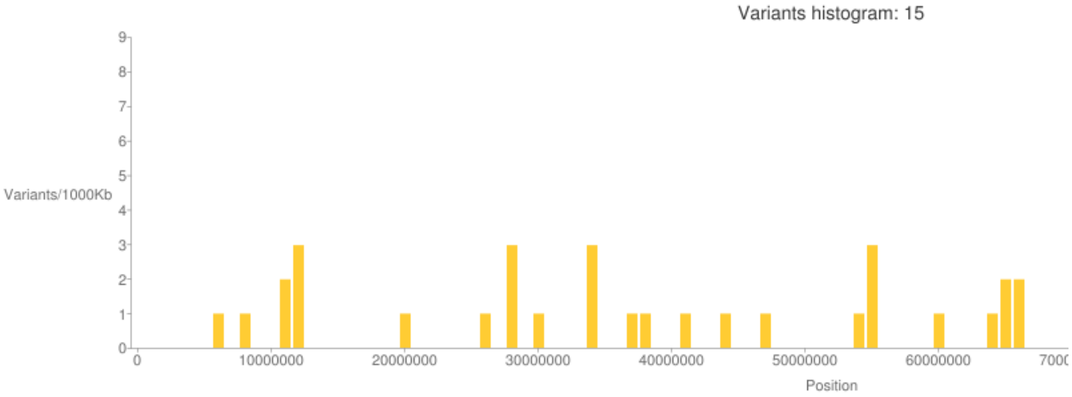

15, Position,0,1000000,2000000,3000000,4000000,5000000,6000000,7000000,8000000,9000000,10000000,11000000,12000000,13000
15,Count,0,0,0,0,0,0,1,0,1,0,0,2,3,0,0,0,0,0,0,1,0,0,0,0,0,1,0,3,0,1,0,0,0,3,0,0,1,1,0,0,1,0,0,1,0,0,1,0,0,0,0,0,0,1,3,0,0,0,0,1,0,0,

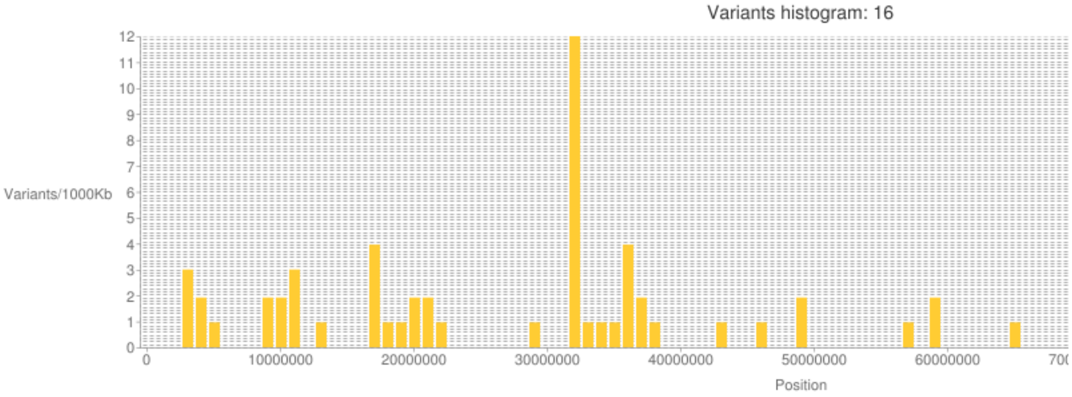

16, Position,0,1000000,2000000,3000000,4000000,5000000,6000000,7000000,8000000,9000000,10000000,11000000,12000000,13000
16,Count,0,0,0,3,2,1,0,0,0,2,2,3,0,1,0,0,0,4,1,1,2,2,1,0,0,0,0,0,0,1,0,0,12,1,1,1,4,2,1,0,0,0,0,1,0,0,1,0,0,2,0,0,0,0,0,0,0,1,0,2,0,0,0

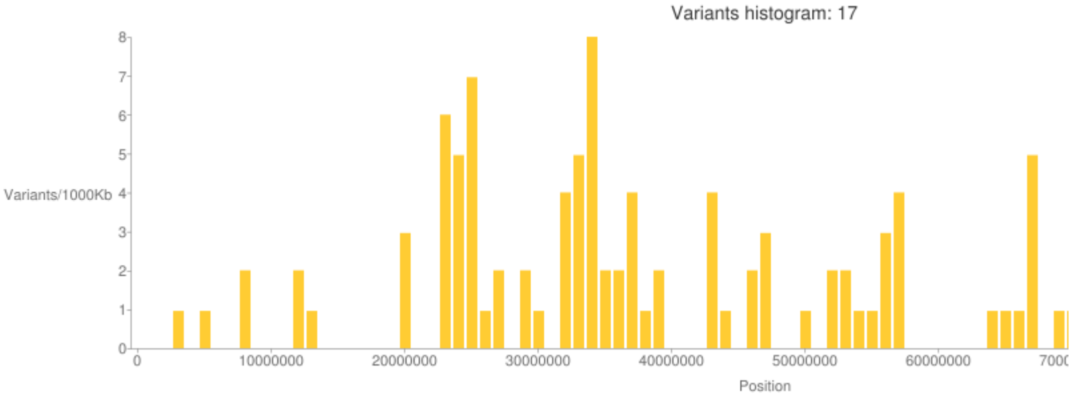

17, Position,0,1000000,2000000,3000000,4000000,5000000,6000000,7000000,8000000,9000000,10000000,11000000,12000000,13000
17,Count,0,0,0,1,0,1,0,0,2,0,0,0,2,1,0,0,0,0,0,3,0,0,6,5,7,1,2,0,2,1,0,4,5,8,2,2,4,1,2,0,0,0,4,1,0,2,3,0,0,1,0,2,2,1,1,3,4,0,0,0,0,0,

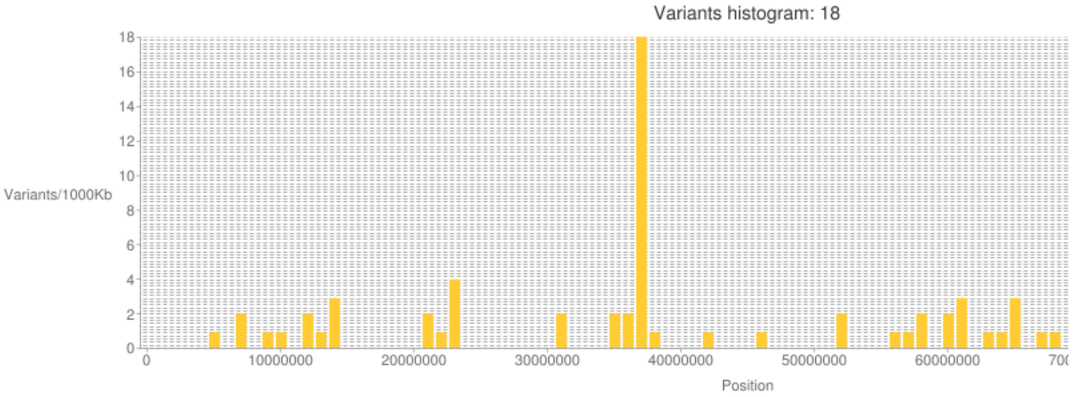

18, Position,0,1000000,2000000,3000000,4000000,5000000,6000000,7000000,8000000,9000000,10000000,11000000,12000000,13000
18,Count,0,0,0,0,0,1,0,2,0,1,1,0,2,1,3,0,0,0,0,0,2,1,4,0,0,0,0,0,0,2,0,0,0,2,2,18,1,0,0,0,1,0,0,0,1,0,0,0,0,0,2,0,0,0,1,1,2,0,2,3,0

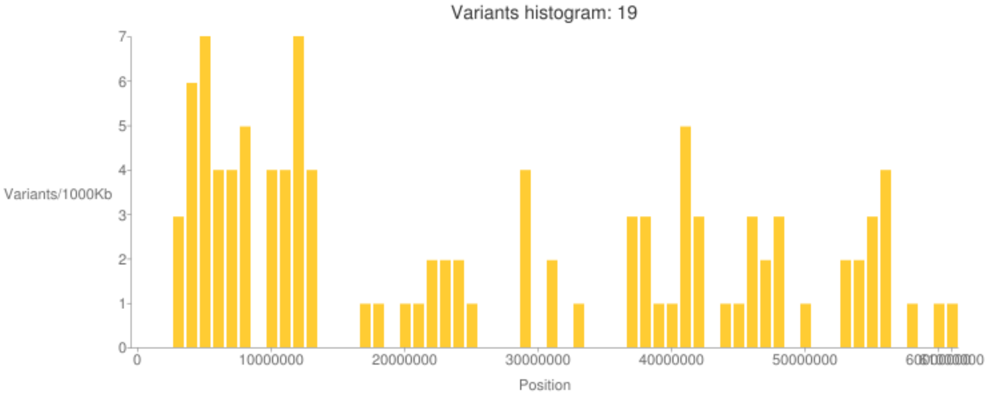

19, Position,0,1000000,2000000,3000000,4000000,5000000,6000000,7000000,8000000,9000000,10000000,11000000,12000000,13000
19,Count,0,0,0,3,6,7,4,4,5,0,4,4,7,4,0,0,0,1,1,0,1,1,2,2,2,1,0,0,0,4,0,2,0,1,0,0,0,3,3,1,1,5,3,0,1,1,3,2,3,0,1,0,0,2,2,3,4,0,1,0,1,1

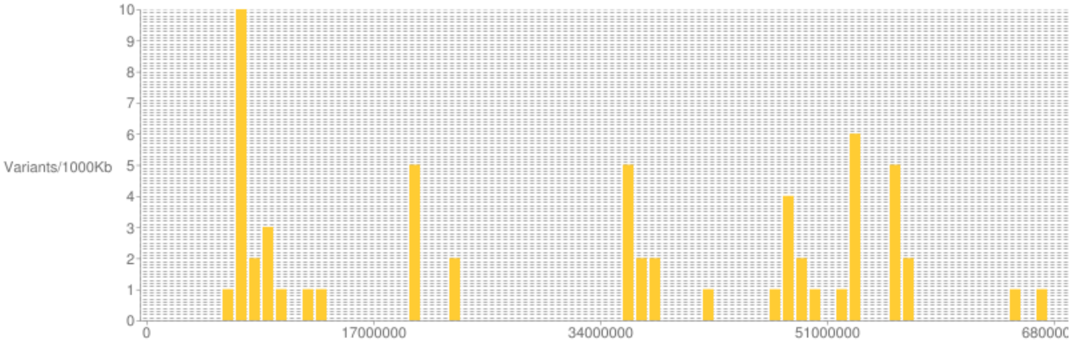

X, Position,0,1000000,2000000,3000000,4000000,5000000,6000000,7000000,8000000,9000000,10000000,11000000,12000000,130000
X,Count,0,0,0,0,0,0,1,10,2,3,1,0,1,1,0,0,0,0,0,5,0,0,2,0,0,0,0,0,0,0,0,0,0,5,2,2,0,0,0,1,0,0,0,0,1,4,2,1,0,1,6,0,0,5,2,0,0,0,0,0,

Details by gene

Here you can find a tab-separated table.
